# Indo-European cereal terminology suggests a Northwest Pontic homeland for the core Indo-European languages

**DOI:** 10.1371/journal.pone.0275744

**Published:** 2022-10-12

**Authors:** Guus Kroonen, Anthony Jakob, Axel I. Palmér, Paulus van Sluis, Andrew Wigman

**Affiliations:** 1 Leiden University Centre for Linguistics, Leiden University, Leiden, The Netherlands; 2 Department of Nordic Studies and Linguistics, University of Copenhagen, Copenhagen, Denmark; Kiel University, GERMANY

## Abstract

Questions on the timing and the center of the Indo-European language dispersal are central to debates on the formation of the European and Asian linguistic landscapes and are deeply intertwined with questions on the archaeology and population history of these continents. Recent palaeogenomic studies support scenarios in which the core Indo-European languages spread with the expansion of Early Bronze Age Yamnaya herders that originally inhabited the East European steppes. Questions on the Yamnaya and Pre-Yamnaya locations of the language community that ultimately gave rise to the Indo-European language family are heavily dependent on linguistic reconstruction of the subsistence of Proto-Indo-European speakers. A central question, therefore, is how important the role of agriculture was among the speakers of this protolanguage. In this study, we perform a qualitative etymological analysis of all previously postulated Proto-Indo-European terminology related to cereal cultivation and cereal processing. On the basis of the evolution of the subsistence strategies of consecutive stages of the protolanguage, we find that one or perhaps two cereal terms can be reconstructed for the basal Indo-European stage, also known as Indo-Anatolian, but that core Indo-European, here also including Tocharian, acquired a more elaborate set of terms. Thus, we linguistically document an important economic shift from a mostly non-agricultural to a mixed agro-pastoral economy between the basal and core Indo-European speech communities. It follows that the early, eastern Yamnaya of the Don-Volga steppe, with its lack of evidence for agricultural practices, does not offer a perfect archaeological proxy for the core Indo-European language community and that this stage of the language family more likely reflects a mixed subsistence as proposed for western Yamnaya groups around or to the west of the Dnieper River.

## 1. Introduction

The puzzle of Indo-European origins is both an age-old and an ever topical problem. With the recent emergence of palaeogenetic methods the current mood is that the debate on the Indo-European homeland, which for several decades was dominated by a rivalry between the Steppe Hypothesis [1–3] and the Anatolia Hypothesis [4], has been decided in favor of the former. Initial genetic studies confirmed that agriculture indeed was mainly driven by demic rather than cultural diffusion [5], thus offering support for the proposed spread of a linguistically homogenous population from the Near East to Europe. However, subsequent genetic studies revealed large-scale late Neolithic gene flow from the pastoralist Yamnaya culture [6–8], a population movement that had been proposed as a driving factor for the Indo-European linguistic dispersal long before DNA had been discovered [9]. Nevertheless, the general optimism about the alignment of genetic, archaeological and linguistic scenarios on the Indo-European homeland cannot detract from the fact that two important problems remain [10].

First of all, many of the details of the linguistic fragmentation of the Indo-European speech community, i.e. the exact phylogenetic model, are still unclear. While there is relative consensus on the basal status of the Anatolian branch, leading to the formulation of the Indo-Anatolian Hypothesis [11:30], the situation beyond the Anatolian split is more blurred. Tocharian, too, is often held to be relatively archaic, i.e. the second branch to split off, but it has alternatively been assigned to the so-called core Indo-European group, consisting of the European branches and Indo-Iranian [12]. Within core Indo-European, various rival models exist, including primarily those prioritizing a Graeco-Indo-Iranian (“Graeco-Aryan”) subnode versus a Balto-Slavo-Indo-Iranian (“Indo-Slavic”) subnode, with Albanian and Armenian as their satellites. Without a generally established phylogeny, the identification of suitable archaeological and genetic proxies for the prehistoric locations and movements of the various Indo-European speech communities, itself a highly challenging endeavor, is all the more treacherous.

The second, here central problem concerns the linguistic reconstruction of Proto-Indo-European economy. The pastoralist elements in the lexicon, including terminology related to the herding of sheep and cattle, are universally acknowledged. The field of Indo-European studies has traditionally been more divided, however, over how much Proto-Indo-European vocabulary can be reconstructed for the cultivation of plant domesticates, particularly cereals. According to Schrader [9], Proto-Indo-European speakers practiced a relatively pure form of pastoralism. This position was called “exaggerated”, however, by Childe [13:90], who assumed that Indo-Europeans “occasionally stooped to cultivate the soil by rude and primitive methods” [13:88–9]. On the other side of the spectrum, the Indo-Europeanist Hirt [14; 15] strongly argued for a fully agrarian Indo-European society. Supporters of both sides have persisted into the twenty-first century: while some postulate a complete lack of agricultural terminology in Proto-Indo-European [16], others admit a wider range of terms [17; 18:7–8].

The controversy around Proto-Indo-European agriculture for a large part derives from differences in the methods used for linguistic reconstruction and ties back into the first problem of Indo-European phylogeny. In the traditional, perfect starburst model, where all branches are equally distantly related, any term that occurs in as few as two branches must be dated back to the protolanguage. Hirt thus arrived at a multitude of agricultural terms, many based on cognate sets only found in the European languages, and assumed that these terms were lost in Indo-Iranian. In a more stratified model, in which the split between the European and Asian branches (i.e. Indo-Iranian and Tocharian) is primary, only terms with continuants in both can be accepted for Proto-Indo-European. According to the latter criterion, Schrader accepted a more limited number, assuming that many of the terms exclusive to the European languages were acquired in Europe after the Indo-Iranian split. These different approaches, one maximalist, the other minimalist, produce highly divergent results.

Moreover, the twentieth century discoveries of Tocharian and Anatolian have had important repercussions for the debate. However, the addition of these languages has magnified the differences rather than resolved them, again due to disagreement on the methodology. Using the starburst phylogenetic model, the addition of evidence from Anatolian and Tocharian, especially when admitting a certain laxness on the formal and semantic side, leads to a substantial increase in the number of proposed lexical comparisons [19–21]. In a structured phylogenetic model, on the other hand, it follows from the basal character of especially Anatolian that reconstructions without cognates in this branch should only be accepted for core Indo-European [16]. In practice, however, a hybrid model has emerged. Terms are granted “Indo-European” status when they *either* are found in a European and an Asian branch, *or* in Anatolian and at least one other branch [10]. The resulting method produces a significant corpus of phylogenetically ambiguous terms related to agriculture (see Table 1).

**Table 1:** Proposed Indo-European agricultural terms found in at least one European and one Asian language.

*ses(i)ós ‘± grain’	*meiǵ^h^- ‘± grain’	*h_3_ekéteh_a_- ‘harrow’
*yéwos ‘±grain,? barley,? wheat’	*h_2_eḱstí- ‘awn’	*seh_1_- ‘sow’
*ǵrh_a_nóm ‘± grain,? barley’	*h_2_éreh_2_- ‘weed/rye’	*wers- ‘thresh’
*ǵ^h^resd^h^i- ‘± grain’	*ālu- ‘esculent root’	*melh_2_- ‘grind’
*b^h^ars- ‘± grain’	*keres- ‘millet’	*peis- ‘grind’
*d^h^oh_x_néh_2_- ‘± grain’	*pano- ‘millet’	*h_2_el- ‘grind’
*drh_x_weh_2_- ‘± grain’	*kāpos ‘field’	*srpo/eh_2_- ‘sickle’
*h_2_ed- ‘± grain’	*h_2_érh_3_ye/o- ‘plough’	*g^w^réh_a_won ‘quern’
*h_2_elb^h^it- ‘± grain,? barley’	*g^h^el- ‘plough’	

Table reproduced from Mallory [10].

The problem now becomes apparent, since the postulation of many agricultural terms does not confirm, but rather challenges the current consensus on the Indo-European homeland [10]. Both in the Steppe Hypothesis and the revised Anatolia Hypothesis [22], the Bronze Age Yamnaya culture of South Russia plays a central role. Under the Steppe Hypothesis, the dispersals of the core Indo-European branches are associated with the expansion of the Yamnaya pastoralists from the Pontic-Caspian Steppe, whereas the Anatolian branch is thought to have migrated to Anatolia from the pre-Yamnaya culture of Sredny Stog [3; 23]. While the original Anatolia Hypothesis sought to overlay the entire Indo-European dispersal onto the spread of farming from Anatolia, a version still maintained by some [24], a modified version envisages the Yamnaya culture as a secondary center of spread for all non-Anatolian branches [22]. Both of these scenarios are problematic if we assume a wide variety of agricultural terms for core Proto-Indo-European, for the simple reason that the evidence for cereal cultivation east of the Dnieper, where the Yamnaya culture emerged [3:317 ff.; 25], is highly dubious until the Late Bronze Age [26:152]. This problem is further underlined by the southern Siberian Afanasievo culture (3300–2500 BCE), with its close genetic ties to the Yamnaya population [7], as no unambiguous evidence for cultivated grains has been identified there so far [27].

A widespread position among steppe archaeologists used to be that Yamnaya societies were involved in ‘sporadic agriculture’ [28:144; 29; 30:276]. From a cross-cultural perspective, it is conceivable that mobile Yamnaya pastoralists practiced agriculture in the river valleys, as is the case for modern nomadic groups inhabiting drylands [26:151–4]. Similar to the later Catacomb culture, parts of the population, perhaps a mobile elite, may have seasonally pastured their cattle on the steppe, while other parts were more sedentary and remained in the river valleys year round [31:194; 32:905]. Traditionally, the presence of stone hoes, mattocks, sickles and grinding stones has been taken as archaeological proof of cereal cultivation [33:71; 34:54], next to cereal and chaff impressions in pottery and daub. Cereal impressions have been reported from the late, western Yamnaya in the Lower Dniester [3:320; 35:120] and from the walled Skelya Kamenolomnya site [36:15].

However, the evidence for cultivation has been reappraised in recent times. Reaping knives can be used for the harvesting of wild plants [37:244] and stone grinding implements have been known since the Palaeolithic for preparing flour from wild grass seeds [38]. The interpretation of cereal imprints can be problematic due to difficulties in dating pottery and challenges in discerning cereal imprints from those of wild seeds with the naked eye. More reliable data comes from macrofossils, i.e. carbonized cereal seeds, especially when they can be directly radiocarbon dated. However, no macrofossils are currently known from Yamnaya sites [37:234; 39:144]. The insignificance of cereals in the diet is further supported by the absence of dental caries from Yamnaya individuals [40:169–71]. Since at least the Yamnaya populations east of the Don may have been fully mobile [41; 42], possibly residing in wagons [3], their lifestyle would have left little opportunity for cultivation.

In conclusion, although archaeologists traditionally do not agree on the question of whether agriculture was practiced by steppe pastoralists, i.e. whether it was practiced sporadically, or in fact, not at all, current consensus appears to be leaning toward a negative answer [43]. Given these increasingly pessimistic results, the assumption that Proto-Indo-European had a wide range of terms for cereal cultivation and processing is not unambiguously consistent with the Steppe Hypothesis. It in fact presupposes an economy in which cereal cultivation played a much greater role than a purely pastoralist lifestyle would allow for. Thus, we are faced with a paradox: we cannot assume that the (core) Indo-European speech community possessed an elaborate set of terms referring to sedentary agriculture, while at the same time endorsing the early Yamnaya culture, with its roots in the Volga-Don steppes, as an archaeological proxy. Despite the genetic confirmation of the Yamnaya expansion as a suitable vector for the spread of the (core) Indo-European languages, the conclusion must be that either the reconstruction of Proto-Indo-European farming vocabulary is flawed or the Steppe Hypothesis is incomplete.

## 2. Methods

To address the apparent contradiction between the linguistic reconstruction of Proto-Indo-European subsistence and the archaeologically documented Yamnaya economy, we here reassess the linguistic evidence on Indo-European cereal cultivation in order to establish to what extent it is in conflict with the archaeological record of the Pontic Region. For this purpose, we offer an etymological corpus of all previously proposed Indo-European lexical comparisons that a) have cognates in at least two Indo-European branches and b) attest semantics related to cereal cultivation and processing. To evaluate this corpus, we assess 1) the formal and 2) the semantic characteristics of the involved lexemes, as well as 3) the position in the phylogeny to which they can be dated. Formally accepted etymologies are those that are based on lexical comparisons whose cognates conform to established sound changes. These etymologies are left unmarked in the corpus. Formally questionable and rejected comparisons are indicated with a question mark and a dagger (†) respectively. Next to the formal analysis, we analyze the semantic details of each of the etymologies to establish whether or not they truly are related to cereal use.

Furthermore, we systematically evaluate 4) where in the phylogeny the involved formal reconstructions arose and where they can be shown to have possessed or acquired a meaning associated with cereal use. Reconstructions and meanings that are found in Anatolian and any other branch are considered ‘basal Indo-European’ or ‘Indo-Anatolian’. When present in at least one European and one Asian branch, these features are considered ‘core Indo-European’. Reconstructions and meanings that are exclusively found in two or more European branches are considered ‘Euro-Indo-European’, ‘dialectal European’ or simply ‘European’. We define Greek, Albanian, Balto-Slavic, Germanic, Italic and Celtic as European branches and Tocharian and Indo-Iranian as Asian branches while remaining agnostic about the status of Armenian.

The resulting stratified corpus is used here to establish the nature of the basal and core Indo-European economies as well as their main differences. The combined result is matched against archaeologically documented economies that have been proposed for Late Eneolithic and Early Bronze Age steppe groups, to see how the linguistic evidence correlates with the Steppe Hypothesis and to what extent this hypothesis can be maintained. Finally, we employ this corpus to clarify the phylogeny of the Indo-European language family, including the positions of Tocharian and Indo-Iranian.

## 3. The data

### 3.1. Indo-European terms accepted by Mallory

?*b^**h**^**ar-(e)s-** (*bhares- [44:111]; *b^*h*^*árs*, gen. *b^*h*^*arés(o)s*? ‘barley’ [18:51]; *bhars- [19:57]; *b^*h*^*ars* ‘± grain’ [10]): OCS *brašьno* ‘food’, Ukr. *bórošno* ‘flour’, Sln. *brášno*, *brašnọ̑*, SCr. *brȁšno* ‘flour, food’ < *borš-ьno-; Go. *bariz*-*eins* a. ‘barley-’, ON *barr* m. ‘grain, barley’, OE *bere* m. ‘barley’ < PGm. *bariz-; Lat. *far*, gen. *farris* n. ‘husked wheat, emmer; grain, flour’, Umbr. *far* ‘flour, meal’ < PIt. *fars-

This European word is traditionally reconstructed as a PIE *s*-stem *b^*h*^*ar*-*(e)s*-, with *a in the root and suffixal ablaut found between Lat. *far*, PSl. *borš- < *b^*h*^*ar*-*s*- and Go. *bariz*-, ON *barr*, OE *bere* < *b^*h*^*ar*-*es*-. A proposed Iranian cognate, Oss. I *bur*-*xor*, D *bor*-*xwar* ‘proso millet’ [19:57; 21:54], is phonologically incompatible, as the Ossetic vocalism points to PIr. *au.

Indo-European *s*-stems typically have *e*- or zero grade in the root, not *a*, even if this vowel is accepted as a (marginal) PIE phoneme. For Lat. *far*, *a*-vocalism can be avoided by postulating that PIt. *far-*os*, *-*es*- < *b^*h*^*r̥H*-*os*, *-*es*-, with the zero grade of a laryngealic root and regular assimilation of the final syllable. PSl. *bъrъ, cf. Ru. *bor*, Pol. *ber*, SCr., Sln. *bȃr* m. ‘(foxtail) millet’, has been derived from the same protoform [19:86; 45:369]. However, Umbr. *farsio* ‘*farreum*’ < PIt. *fars-*ejo*- cannot be derived from syncopated *fare/os-*ejo*-, as this would have resulted in **farfio, with -*rf*- from secondary *-*rs*- [46:113]. More fatally, the required root *fars- excludes a laryngealic reconstruction *b^*h*^*rH*-*s*-, because this would have developed into **frās-.

Those who do not accept *a as an Indo-European phoneme, have expressed about the Indo-European origin of this word, not least in view of the absence of cognates in the Asian branches [46:113–4; 47:287]. Starting from a donor form *b^*h*^*ars*-, it is possible to account for the corresponding Slavic and Italic forms, and perhaps also for the Germanic form, by assuming that it was incorporated into the *s*-stems within Germanic [48:201]. However, it cannot be excluded that Germanic borrowed the word as *b^*h*^*ares*- or *b^*h*^*aris*-. If correct, the evidence would favor a scenario in which multiple European subgroups, when moving into Europe, independently adopted a cereal term, e.g. *b^*h*^*ar(V)s*-, from an unknown source.

Finally, the appurtenance of some Celtic forms, OIr. *bairgen* f. ‘bread’ < *bare/iginā, W, Corn., Bret. *bara* m. ‘bread; food’ < *barag-, is uncertain, because it requires segmentation of the formation into a root *bar- < *b^*h*^*ar*- and an otherwise obscure velar suffix *-*eg*- [44:108–9; 49:101; 50:B, 9]. British and Goidelic appear to have a different vowel in the suffix, but a single Proto-Celtic reconstruction *bareginā is possible under the assumption of a sound law PC *e > PBr. *a before *ge, *gi [51:134–41] (see under *seǵ^**h**^**-e-tleh**_**2**_**-**).

*d(e)rH-ueh_**2**_**-** (*dr̥̄-*u̯ā* [44:206–11]; *dŕ̥h_*x*_*weh*_*a*_- ‘± grain’ [18:237]; *dr̥̄̆HwaH_*2*_ [19:83]; *drh_*x*_*weh*_*2*_- ‘weed, rye’ [10]): Skt. *dū́rvā*- f. ‘dūrvā grass (*Panicum dactylon*)’ < PIIr. *drH-u̯aH-;? Lith. *dirvà* f. ‘arable field’, Latv. *dìrva* f. ‘id.’ < PB *dirvaʔ; ME *tare* ‘vetch, weed growing in grainfields’, MDu. *tarwe*, *terwe* c. ‘wheat’, Du. *tarwe* ‘wheat’ < PGm. *terwō- or *tarwō-; Gaul. *drāuā (>> Fr. *droue* ‘darnel’), Gallo-Lat. *dravoca* (>> Du. *dravik*), W *drewg*, Bret. *draok*, *dreok* ‘darnel’ < PC *drāu̯(ā/uk)ā

An *uH*-stem to a root *derH- can be identified in at least Germanic, Celtic and Indic, a distribution pointing to an Indo-European origin [52:313]. The Sanskrit form has alternatively been reconstructed as *dr-*uaH*- under the assumption of a change *-*ŕ̥u̯*- > *-*ū́ru̯*- [53:149 fn. 29], but Proto-Celtic *drāu̯(ā/uk)ā [54:148] requires a laryngeal. In view of this, the traditional comparison with Lith. *dirvà*, Latv. *dìrva* f. ‘field’, with its non-acute root, is uncertain [47:288].

A key question concerns the original meaning of the formation, sometimes suggested to be ‘rye’ [10]. Skt. *dū́rvā*- designates a (sacred) wild grass. In Germanic, the related term seems to have been applied to a variety of weeds. The specifically Dutch development into ‘wheat’ is remarkable, but late and unquestionably secondary. In Celtic, *drāu̯ā- referred exclusively to darnel, a wild grass infesting grain fields. Since all certain attestations except the Dutch ones point to a wild grass, this is likely to be the oldest senst.

*d^**h**^**oH-neh**_**2**_**-** (*dhōnā [44:242]; *dhoh_*x*_*néh*_*a*_- ‘grain’ [18:237]; *dhoHnáH_*2*_ [19:39–40]; *d^*h*^*oh*_*x*_*néh*_*2*_- ‘± grain’ [10]): ToA *taṃ*(?), ToB *tāno*, obl. *tāna* f. ‘grain, (sesame, lotus) seeds’ <? PTo. *tānā-; Skt. *dhānā́ḥ* f.pl. ‘roasted grains’, Khot. *dānā*- ‘grain, (sesame, grape) seeds’, Av. *dānō*-*karša*- ‘grain-carrying(?)’, Sogd. *d’n* ‘grain’, MP *d’n* ‘grain, seed’, *šyfšd’n* n. ‘grain of mustard’, NP *dāna* ‘grain, berry, stone (of fruit), seed’, Psht. *daná* ‘grain, kernel, granule’ < PIIr. *d^**h**^**aHnaH(-kaH)-**; Lith. *dúona* f. ‘(loaf of) bread; (bread) rye’, Latv. *duõna* f. ‘(end) slice of bread’ < BSl. *doʔnaʔ

A formation *d^*h*^*oH*-*neh*_*2*_- can be reconstructed on the basis of Indo-Iranian and Baltic. In Baltic, the original meaning appears to have been ‘a cereal’ [55:266], which then shifted to ‘bread’. It has been suggested that the Baltic word is etymologically identical to Latv. *duõna* ‘edge, rim’ < *doh_*2*_-*neh*_*2*_-, and originally meant ‘slice’ [56:258–9], but it seems more likely that the two words merely influenced each other. In Indo-Iranian, the oldest meaning is ‘grain’, but the word also refers to the small seeds of other domesticates, cf. Skt. *dhānaka*- n. ‘coriander’.

The appurtenance of the Tocharian word is uncertain, since its semantics [56:257–9] and inflectional class [57:243] favor a Khotanese source. Likewise, an Iranian origin is plausible for Old Turkic *tana* ‘grain of coriander’ [58:515] and Mong. *tana* ‘(mother of) pearl’, even if Tocharian served as an intermediate language [59:303].

Other suggested cognates must be rejected. The connection of Hitt. *dannaš*- ‘a type of bread’ [60] is doubtful, as it would have to be interpreted as a denominal *s*-stem *d^*h*^*H*-*n*-*h*_*2*_-*es*-, whose ablaut is derivationally problematic. Middle Armenian *don* ‘bread’ is best explained, despite Martirosyan [61:241–3], as a loan from Urartian, cf. Hur. *tuni* ‘a kind of bread’ (whence also Hitt. *dūni*- ‘a pastry’), because it does not show the expected change of *oN > *uN*. Finally, Alb. *duaj* n.pl. ‘sheaves of grain’, connected by Orel [62:16], is more likely to be derived from *deh_*1*_-*mon*-, cf. Skt. *dā́man*- ‘cord, rope’ [63:149] or *d^*h*^*eh*_*1*_-*mon*-, cf. Gk. θημών ‘heap’.

In sum, only the Indo-Iranian and Baltic forms remain from the aforementioned comparanda. Whether or not this formation can be assigned to the core Indo-European level depends on the preferred phylogenetic model, i.e. traditional or Indo-Slavic. In the latter case, the formation would only have existed in one of the shallowest subclades.

*ǵ^**h**^**(e)rs(d)-** (*ĝherzd(h), Gen. *ĝhr̥zd(h)-*es*; *ĝherzdā* [44:446]; *ĝ^*h*^*resd*^*h*^*(i)*, gen. *ĝ^*h*^*rsd*^*h*^*ós* ‘barley’ [18:51]; *ĝhersd(h): *ĝhrī́d(h) [19:55–6]; *ĝ^*h*^*resd*^*h*^*(i)* ‘± grain’) [10]:? Hitt. *karaš* n. ‘wheat, emmer-wheat’; Alb. *drithë* f. ‘cereals, grain’ < PAlb. *driδ-(?); OS *gersta*, OHG *gersta* f. ‘barley’ < PGm. *gerstō-; Lat. *hordeum* n. ‘barley’ < PIt. *χord-ejo-

An element *ǵ^*h*^*ersd*- (not *ǵ^*h*^*ersd*^*h*^-) is supported by Italic and Germanic, as well as potentially by Anatolian and Albanian. The old comparison with NP *zurd* ‘a kind of millet’, dial. *ǰurda* ‘grain’ [19:55, 87; 64:140; 65:571; 66] must be abandoned in view of additional Iranian evidence for a reconstruction *(H)iau(H)a-*Hart*- ‘milled grain’ [21:54; 67:22].

Regarding the Albanian form, one obstacle to deriving *drithë* from *ǵ^*h*^*r̥sd*- is that palatovelars otherwise appear to have been depalatalized by a following resonant [68:1745]. This could be an argument in favor of the alternative comparison with Gk. κρῖ n., κριθή f. ‘barley’, but the problem can be resolved by assuming that syllabic *r̥ did not cause depalatalization [69:277]. A second issue concerns the origin of Alb. *th*. One solution is that it is regular from PIE *sd [70:145, 149], in which case *drithë* may straightforwardly be derived from PAlb. *drisdā < *ǵ^*h*^*rsd*-*eh*_*2*_-. Alternatively, we can assume that *sd and *sd^*h*^ both became *dh*, but that it was devoiced word-finally [71:261]. The *th* of *drithë* would then have to be analogical, i.e. leveled from a PAlb. paradigm *driθ, pl. *driδā [72:257].

Much of the formal variation found across the branches can be accounted for by starting from a neuter root noun. The Germanic formation implies a preform *ǵ^*h*^*ersd*-*eh*_*2*_- resembling a collective. Lat. *hordeum* also appears to be a collective formation, but the suffix *-*ejo*- is isolated to Italic and doubtlessly late. Alb. *drithë* may continue a paradigm *ǵ^*h*^*rsd*, pl. *ǵ^*h*^*rsd*-*eh*_*2*_. From this perspective, it is also possible to compare Hitt. *karaš* [73:60]. However, the connection hinges on the assumption of either a root extension *-*d*- in core Indo-European [74:63–5] or (regular) loss of the dental in case forms in which it was in word-final position [75:444].

?*ǵ^**h**^**olH-o-** (*ĝhel- *2* ‘schneiden’?? [44:434];? *ǵhel- ‘plow’ [18:435]; *g^*h*^*el*- ‘plough’ [10])

On the basis of Skt. *hala*- ‘plow’ and Arm. *jlem* ‘make furrows’, a verbal root *ǵ^*h*^*el*- ‘plow’ has been hypothesized, but the etymology is problematic.

First of all, the reconstructed meaning ‘plow’ appears to have been cherry-picked from the broader semantic range exhibited by its alleged continuants, viz. MW *geleu* ‘knife’ < PC *gelVu̯- (for the suffix, cf. MW *cleddeu* ‘sword’ and W *neddau* ‘adze’), OE *gielm* ‘sheaf’, WFri. *galm* ‘armful’ < *gelma- [76:5–8] and Go. *gilþa* m. ‘sickle’ < *gelþan-. The root is generally reconstructed with the more basic meaning ‘cut’ [44:434].

Second, a shared protoform can strictly speaking only be reconstructed for Skt. *hala*- ‘plow’ and Arm. *joł* ‘stick’, i.e. by assuming a potentially shared and inherited *o*-*stem* *ǵ^*h*^*olH*-*o*-. The hapax Arm. *jlem* ‘furrow’, if reliable, would rather presuppose an ablauting variant *ǵ^*h*^*ēl*- or *ǵ^*h*^*ōl*- [61:435]. Even if the reconstruction of a term *ǵ^*h*^*olH*-*o*- is justified, the involved semantics suggest that it originally meant ‘stick’ [44:434] and acquired the meaning of an agricultural implement only secondarily, in Indic. However, Skt. *hala*- has alternatively been interpreted as a loan from a non-Indo-European source [77:2, 808].

*ǵrH-no- (*ĝr̥-*nóm* [44:390–1]; *ĝrh_*a*_*nóm* ‘grain’ [18:236]; *ĝr̥Hnóm [19:43, 116–7]; *ĝrh_*a*_*nóm* ‘± grain,? barley’ [10]):? Psht. *zə́ṇai* ~ *zə́ṛai* m. ‘seed, pit; stone of a fruit; core, nucleus’ < PIIr. *j́rH-na(-ka)-(?); Lith. *žìrnis*, Latv. *zir̃nis* m. ‘pea’, OPru. *syrne* ‘grain’ < PB *žirʔni(o)-; OCS *zrъno*, Ru. *zernó*, SCr. *zȑno* n. ‘grain’ < *zьrno; Go. *kaurn* n. ‘grain, seed, wheat’ < PGm. *kurna-; Lat. *grānum* n. ‘grain, seed, kernel’ < *grāno-; OIr. *grán* n. ‘grain’, MW *grawn* n. ‘grain, cereal, seed; berries’ <? PC *grāno-

The formation *ǵrH-*no*- is relatively widely attested, with cognates in many European branches as well as potentially in Iranian. The appurtenance of the Celtic form is uncertain, as it may be a Latin loan. The derivation of Oss. *ʒærna* (“dzärná”) ‘frumenty’ from *ǵrH-*no*- [78:47] cannot be maintained, since *ʒ* can only go back to PIIr. *ǰ.

The primary meaning of *ǵrH-*no*- was likely ‘granule’ [16:40; 79:23] rather than “Reibefrucht” [44:390–1]. This meaning is attested directly in Italic and Germanic. It seems to have evolved first into ‘seed’ in core Indo-European, and then into ‘cereal’ in Germanic, Italo-Celtic and Balto-Slavic. Both meanings coexist in the former two branches. The isolated Pashto forms *zə́ṇai* ~ *zə́ṛai*, if indeed continuing PIIr. *j́rH-*na*-*ka*- < PIE *ǵrH-*no*- [80:102; 81:103], preserve a less evolved semantic stage, i.e. ‘seed (of any plant)’ rather than ‘grain seed’. However, it should be noted that the alternation of -*ṇ*- with -*ṛ*- is difficult to account for. Psht. -*ṇ*- is the regular outcome of *-*rn*-, although *waṛə́i* ‘wool’ < *HurH-*na*- and esp. the variation of *maṇá* ‘apple’ ~ *maṛa*-*γúne* ‘colocynth, bitter apple’ (lit. “apple-like”) < *amarnā- provide some support for an additional (conditioned?) outcome -*ṛ*-. Psht. -*ṛ*- usually continues *-*rt*- or *-*rd*-.

Etymologically, *ǵrH-*no*- can be derived from the root *ǵerH- ‘crumble, scatter’. Traditionally, this root has been equated with *ǵerh_*2*_- ‘age, mature’, cf. Skt. *jár*^*i*^ ‘age’, OCS *zьrěti* ‘ripen’, through a meaning “aufgerieben werden, von Alter oder Krankheit” [44:390–1]. It cannot be excluded, however, that there were originally two unrelated roots: 1) *ǵerh_*2*_- ‘age’ and 2) *ǵerH- ‘become ground’ [82:165 fn. 1]. The root is further found in Lat. *glārea* f. ‘gravel’: if dissimilated from *grārea, this formation may have been derived from an unattested adjective *glāro- < *ǵrH-*ro*- ‘grainy’ [83:I, 605–6]. More straightforward cognates exist in Celtic and Germanic: W *gro* ‘pebbles, gravel, sand’, OCo. *grou* ‘sand’ < PC *grāu̯ā (whence possibly Fr. *grève* f. ‘riverbank, shore’, Cat. *grava* f. ‘gravel’) < *ǵrH-*ueh*_*2*_- [84] and ON *kjarni*, OHG *kerno* m. ‘core, kernel’ < PGm. *kernan- < *ǵerH-*n*-*on*-. Finally, a verbal attestation can be seen in Lith. *žìrti* ‘fall, scatter’.

*g^**w**^**r(e)h**_**2**_**-uon-** (*g^*w*^*r̥̄*-*nu*-, *g^*w*^*rāu̯*-*ō(n)*- ‘Mühle’ [44:476–7]; *gréh_*a*_-*u̯*-*on*- ~ *g^*w*^*érh*_*a*_-*n*-*u*- ‘quern’ [18:237]; *g^*w*^*réh*_*a*_*won*- ‘quern’ [10]): ToA kärwañi*, ToB kärweñe* ‘stone, rock’ < PTo. *kərwen-; Skt. *grā́van*- m. ‘(pressing-)stone, rock’ < PIIr. *graH-uan-; Go. *asilu*-*qairnus* m. ‘donkey mill’ < PGm. *kwernu-; OIr. *bráu*, *bró* f. ‘millstone, quern’, W *breuan* f. ‘quern’ < PC *grāu̯on-; Arm. *erkan* ‘millstone’ < PArm. *kra(u̯a)n-; Lith. *gìrnos*, Latv. *dzir̃nas* f.pl. ‘quern’ < PB *girʔnaʔ-; Latv. *dzir̃nus* f.pl. ‘quern’, OPru. *girnoywis* ‘quern’ < PB *giʔrnuʔ-; OCS *žrъny* f. ‘millstone’, Ru. *žërnov* m. ‘millstone’, SCr. *žrvȃnj* m. ‘quern’ < PSl. *žьrny

A formation *g^*w*^*reh*_*2*_-*uon*- can be reconstructed on the basis of Tocharian, Indic and Celtic. Armenian could continue *g^*w*^*reh*_*2*_-*un*-, through PArm. *krau̯an- and regular loss of the labial glide, although the alternative reconstruction *kran- < *g^*w*^*reh*_*2*_-*n*- cannot be rejected [61:266]. Armenian, therefore, potentially clusters with the Germanic and Balto-Slavic forms continuing *g^*w*^*erh*_*2*_-*nu*- and *g^*w*^*rh*_*2*_-*nuH*-, respectively [85:566]. These variants appear to be based on a protoform in which the suffix *-*u̯n*- was metathesized to *-*nu*-, a development which may be compared with the regular metathesis *-u̯r- > *-ru- between consonants [86:260; 87:161–2]. It can accordingly be hypothesized that the paradigm originally featured some metathesized forms, e.g. nom. *g^*w*^*réh*_*2*_-*nu*-*s*, gen. *g^*w*^*rh*_*2*_-*un*-*ós*. On the basis of the oblique cases, several branches innovated a new strong stem *g^*w*^*reh*_*2*_-*uon*-. The *Schwebeablaut* of Germanic *g^*w*^*erh*_*2*_-*nu*- may have been introduced analogically after the zero-grade *kwurn- < *g^*w*^*rh*_*2*_-*n*-.

Concerning the semantics, it is generally assumed that the original meaning of the word was ‘stone’ [88:II, 50–1]. Winter [89:187] made the observation that the preservation of this meaning in Tocharian as opposed to the development into ‘grinding stone’ or ‘quern’ in the European branches can be seen as an archaism, and provides an argument for an early Tocharian split. Interestingly, Indic takes up an intermediate position between Tocharian and the European branches, as Skt. *grā́van*- has both the meanings ‘grinding implement (for soma)’ and ‘stone’ (also cf. Pk. *gāva*- m. ʻstone, mountainʼ).

As an archaeological caveat, stone grinding tools cannot be interpreted as exclusive indicators of (domesticated) cereal processing. They are known to have been used for the processing of wild plants and their seeds from the Upper Palaeolithic [38; 90].

?*h_**2**_**ed-o(s)-** (*ades-, *ados- [44:3]; *h_*2*_*ed*- ‘grain, barley’ [18:273]; *H_*2*_*adHor* [19:101–3, 117–8]; *h_*2*_*ed*- ‘± grain’ [10]): Arm. *hat* ‘grain’ < *h_**2**_**ed-o(s)-**; Go. atisk(s*) ‘grainfield’, OHG *ezzisc* m. ‘seeds’ < *atiska-; Lat. *ador*, -*ō̆ris* n. ‘sacral grain, (roasted) spelt’ < PIt. *adō̆s- or *adō̆r-; OIr. gl. *ad* ‘*ador*’ <? PC *ad(-os)-

An *s*-stem *h_*2*_*ed*-*os* has been proposed to be continued by several European branches. This reconstruction works for Lat. *ador*, OIr. *ad* ‘gl. *ador*’ [91:293] and Arm. *hat*, but the Irish and Armenian forms can alternatively be derived from a root noun *h_*2*_*ed*- or an *o*-stem *h_*2*_*ed*-*o*-. No *s*-stem can be postulated on the basis of PGm. *atiska- (as if from *h_*2*_*ed*-*es*-*ko*-), which rather continues an adjective in *-*iska*- [92:188], perhaps in elliptic use, e.g. *atiskaz akraz “seed field”. As a result, the *s*-stem exclusively rests on Lat. *ador*. However, this form is in fact ambiguous as well, and has been derived both from a collective *s*-stem *h_*2*_*ed*-*ōs* [48:25; 93:128] and a collective *r*-stem *h_*2*_*ed*-*ōr* [92].

In support of the latter, Hitt. *ḫattar*, *ḫātar* n. ‘unknown foodstuff, lentils’ has previously been compared [94:220] through a reconstruction *h_*2*_*ed*-*ō̆r* [92; 95]. However, the more frequent variant *ḫattar* rather mandates a protoform with *t. An alternative connection has therefore been proposed with ToA *āti*, ToB ātiyo* n. ‘grass’ < PTo. *ātəyā- < *h_*2*_*et*-*u*-*ieh*_*2*_-, Ru. *otáva* ‘aftermath’ [59:9; 96]. The formally and semantically similar Oss. I *taw*, D *tawæ* ‘aftermath’ is likely a Slavic loan.

Outside Europe, YAv. *āδū*-*fraδāna*- (Y. 65. 1) has been adduced. This hapax was originally glossed as ‘den Eifer, Tatendrang fördernd, mehrend’ [97:322], but this was later modified to ‘abounding in grain’ in view of the similarity to the related Sogd. *ʾʾdwk*, *ʾʾdʾwkh* ‘produce(?), seed grain(?)’ [98:968–9; 99; 100:1–7] < PIIr. *Hād^*(h)*^-*u(*-*kā̆)*-. An unattested Old Persian cognate *ādu-, potentially found in the month name *ādukainaiša*, further appears to have been borrowed by Elamite as *ḫa*-*du*-*iš* ‘revenue, yield, increase’ [101:737–8]. It is not universally accepted that the Iranian formation is related to those found in Europe [102; 103], but if it is, it must continue an *o*-grade *u*-stem *h_*2*_*od*-*u*- [93:128]. Derivation from the root *h_*1*_*ed*- ‘eat’ [100:6–7] is less attractive [104:280].

In conclusion, the reconstruction of an *s*-stem *h_*2*_*ed*-*os*- is possible for, or at least not contradicted, by Italic, Celtic and Armenian. In addition, a *u*-stem to the same root may be identified in Iranian. This can be used as evidence for the postulation of a core Indo-European root *h_*2*_*ed*- that was somehow associated with (domesticated) cereals. It is possible that this root is identical to *h_*2*_*ed*- ‘dry, parch’, cf. Hitt. *ḫāt*-^*i*^ */ ḫat*- ‘dry up, to become parched’ < *h_*2*_*od*-, Gk. ἄζω ‘dry up’ < *h_*2*_*ed*-*ie*- [92]. If correct, the implied semantic specialization can be understood from the fact that hulled wheats need to be parched before they can be dehusked [105:247–8]. However, the practice of parching wild grass seeds is known since the Mesolithic [106; 107] and if the root *h_*2*_*ed*- originally referred to such a practice, a semantic extension to the roasting of cereal grains after the Indo-Anatolian stage would have been natural.

*h_**2**_**e(-h**_**2**_**)i-r-ieh**_**2**_**-** (*ai*-*rā* [44:16]; *h_*2*_*éreh*_*a*_- ‘weed/ryegrass’ [18:7]; *h_*2*_*éreh*_*2*_- ‘weed/rye’ [10]):? Skt. *erakā*- f. ‘reedmace’, Pa. *eraka*-, *era*-° n. ‘reedmace’; Gk. αἶρα f. ‘ryegrass, darnel’ < PGk. *air(i̯)ā-; Latv. *aĩrenes* ‘ryegrass’, dial. *aĩres* f.pl. ‘a kind of weed’ < PBSl. *airiaʔ-

Gk. αἶρα is formally and semantically close to Latv. *aĩres*. The latter served as the basis for the creation of the secondary formation *aĩrenes* with the productive suffix -*ene* [108:I, 284–5]. Both αἶρα and *aĩres* can be derived from a single protoform, viz. *h_*2*_*ei*-*r*-*i(e)h*_*2*_- or—if the underlying acute intonation of the Latvian form can be taken at face value—reduplicated *h_*2*_*e*-*h*_*2*_*i*-*r*-*i(e)h*_*2*_-. Thus, it is possible to assume an inherited PIE formation referring to a wild grass, possibly ryegrass in view of this meaning being attested in both Baltic and Greek.

**Fig 1:**
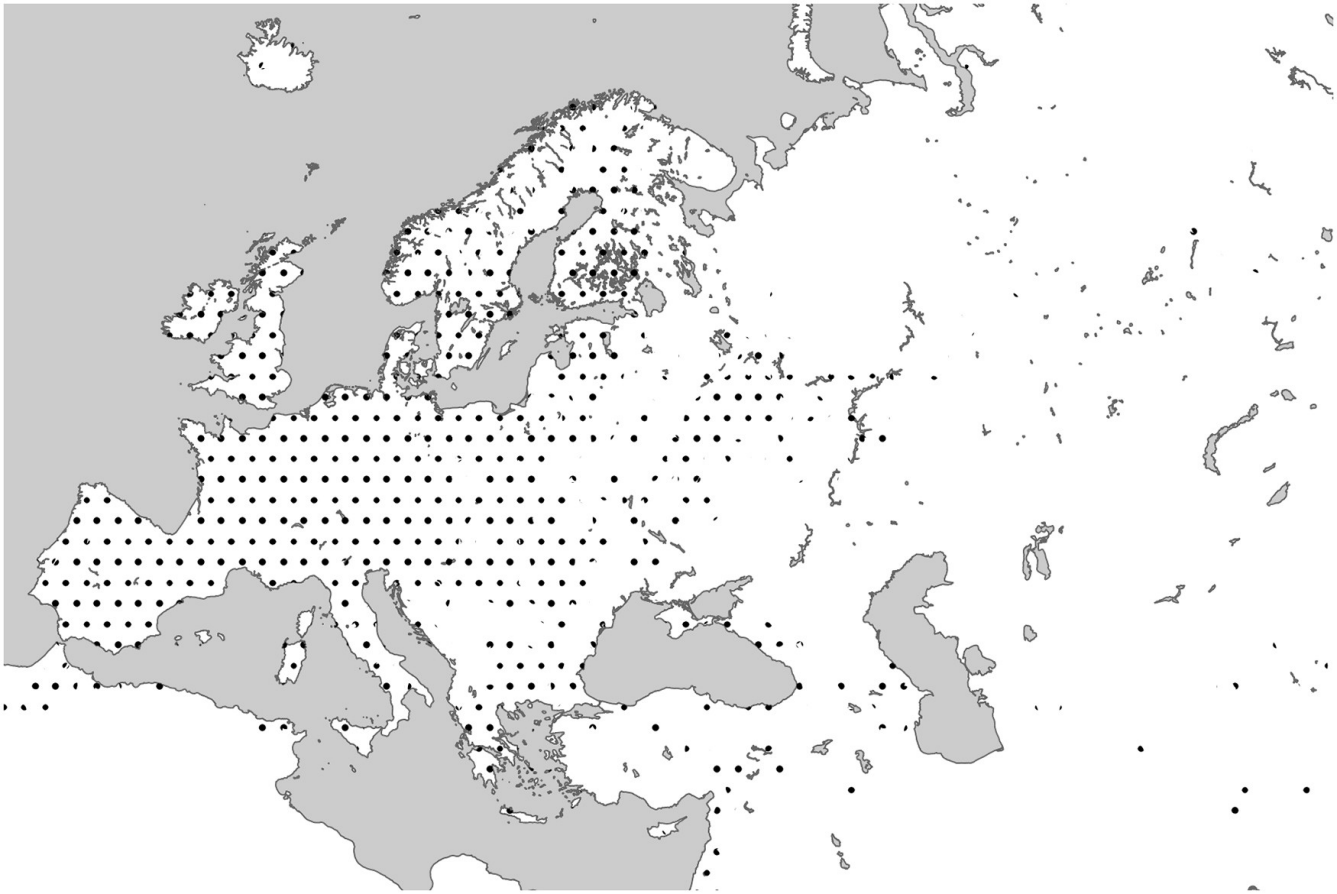
Present-day distribution of perennial ryegrass (*Lolium perenne*). (Data from GBIF.org, https://doi.org/10.15468/dl.4tsemc, visited 7 May 2021).

Cognacy of the traditionally compared Skt. *erakā*- [44:16; 109:12] appears less certain [77:I, 269], but remains a possibility through a protoform PIIr. *Ha(H)ira- < PIE *h_*2*_*e(*-*h*_*2*_*)i*-*ro*- [110:34]. Its meaning has previously been unclear, with proposals ranging between ‘grass’ and ‘watercress’ [111:209 fn. 96], but has convincingly been identied as ‘reedmace (*Typha*)’ [112]. Although semantically more remote, the assumption of a shift from ‘wild grass’ to ‘reedmace’, e.g. through ‘rush’, in the prehistory of Indo-Iranian is difficult to exclude, not least because ryegrass is largely not native to Asia (see Fig 1). On the other hand, there is the possibility of comparing Gk. αἶρα ‘hammer; axe head (Hes.)’. If this is the same word, it would imply an old *Benennungsmotiv* (as in E *reedmace*).

In conclusion, it is possible to reconstruct a (core) PIE term *h_*2*_*e(*-*h*_*2*_*)i*-*r*-*ieh*_*2*_-, perhaps a collective created to a more primary protoform *h_*2*_*e(*-*h*_*2*_*)i*-*ro*-, as potentially supported by the Indic evidence, that originally referred to a reed, rush, sedge or grass. Despite an earlier claim to the contrary [10:149], there are no clear indications that this term originally had an agricultural connotation.

*h_**2**_**eḱ-os-** (*ak̂es-: *ak̂s- [44:18–22]; *h_*a*_*ek̂es*- ‘ear of grain’ [18:237]): ToA āk*, ToB *āke* n. ‘end, tip (of grass)’ < PTo. *ake; Go. *ahs*, ON *ax*, OE *ēar*, OHG *ahar*, *ehir* n. ‘ear of grain’ < *ahiz- ~ *ahsa-; Lat. *acus*, -*eris* n. ‘husks of grain or beans; chaff’ < PIt. *akos-

A PIE *s*-stem with the meaning ‘awn’, ‘husk of grain’ *vel sim*. has been postulated on the basis of Germanic and Latin. This meaning probably does not go back to Proto-Indo-European, however. A possible Tocharian continuant of the *s*-stem, with the meaning ‘tip (of grass)’, appears semantically more primary, not least in view of the likely derivational base *h_*2*_*eḱ*- ‘sharp’. As a consequence, the agricultural connotation of PGm. *ahiz- ~ *ahsa- and Lat. *acus* must have developed secondarily, after the Tocharian split.

The potentially related ToB *āka* ‘millet’ does have an agricultural meaning [113:50]. This form has been interpreted as deriving from a collective *s*-stem *h_*2*_*eḱ*-*ōs* [114:371]. However, the implied derivational pathway appears to be without parallels in Tocharian [56:253–4]. Since *āka* at face value continues PTo. *aka < *h_*2*_*eḱ*-*h*_*2*_, it might be preferable to separate it from the European *s*-stems and instead postulate a root noun *h_*2*_*eḱ*- as the derivational base [59:39–40]. Regardless, if the word was derived from the PIE root *h_*2*_*eḱ*-, the meaning ‘millet’ could have easily developed within Tocharian; compare parallel derivations such as Lat. *pānicum* ‘millet’ from Lat. *pānus* ‘tuft, ear (of millet)’ (see †*pano-).

?*h_**2**_**eḱ-ti-** (*ak̂sti- [44:18–22]; *h_*a*_*ek̂stí*- ‘awn, bristle’ [18:237]; *h_*2*_*ek̂stí*- ‘ear’ [10]):? ToB *āśce* f. ‘head’ < PTo. *aśc-; Lith. *akstìs*, dial. *akštìs* f. ‘thorn, prick’, Latv. *aksts* m. ‘prickle, tip’ < PB *a(k)^**(**^**ś**^**)**^**ti-**; Ru. *ost’* f. ‘awn’, Pol. *ość* f. ‘fishbone, awn, thorn’, Sln. *ǫ̑st* f. ‘point, prick’ < PSl. *ȏstь

A formation *h_*2*_*eḱ*-*s*-*ti*- has been reconstructed for Balto-Slavic and Celtic, but an alternative reconstruction *h_*2*_*eḱ*-*ti*- has been considered as well [115:48]. Whichever is correct, the purported agricultural meaning ‘ear’ is limited to (modern) Slavic, where it is evidently secondary. In Celtic, (M)W *eithin*, OBret. *ethin* ‘furze’ (whence Fr. dial. (Norm.) *hédin* ‘gorse’ [116:XX, 9] < PC *ax(s)tīno- [50:A, 57] appears to be a *Weiterbildung* to PC *ax(s)ti-, but the related OIr. *aittenn* m. ‘furze’ rather points to a PC form *attinno-. Even if the Irish form is a Welsh loan [44:18–22; 117:63], the meaning ‘furze’ is more easily derived from ‘prickle’ than from ‘ear’ and thus further challenges the assumption of an original agricultural association. ToB *āśce* can probably also only be maintained as a cognate by assuming a semantic development from ‘tip’ to ‘head’ [59:61]. In conclusion, although there may have been a formation *h_*2*_*eḱ(*-*s)*-*ti*- in core Indo-European, including Tocharian, the semantic specialization as an agricultural term occurred as late as dialectal Slavic.

*h_**2**_**(e)lb**^**h**^**-it-** (*albhi- [44:29]; *h_*2*_*élb*^*h*^*it* ‘barley’ [18:51]; *albhi [19:58–9]; *h_*2*_*elb*^*h*^*it*- ‘± grain,? barley’ [10]): Gk. ἄλφι, pl. ἄλφιτα n. ‘barley-groats’ < PGk. *alp^**h**^**it-**; Alb. *elb* m. ‘barley’ < PAlb. *albi(t)-

This Greek-Albanian isogloss is without further cognates [63:164–5]. Psht. *orbəša* f. ‘barley’ and similar forms have been compared through a protoform PIr. *arbusa- [18:51; 21:53; 67:367; 118:92]. However, these go back to unrelated PIIr. *arp- [81:10; 119:281], itself highly reminiscent of Turkic *arpa ‘barley’ [120:9]. Without the Iranian cognate, the word acquires a distinctly areal distribution, meaning that it cannot be mechanically projected back into (the oldest phase of) Proto-Indo-European.

Etymologically, the formation *h_*2*_*(e)lb*^*h*^-*it*- can possibly be derived from PIE *h_*2*_*elb*^*h*^- ‘white’, with the suffix *-*it*- as found in PIE *mel-*it*- ‘honey’ [121:136–9] and perhaps in the isolated Hitt. *šeppit(t)*- ‘type of cereal’ [74:158–9; 75:744; 122:27]. Nevertheless, a foreign origin cannot be excluded.

*h_**2**_**erh**_**3**_**-** (*ar(ə)- ‘pflügen’ [44:62–3]; *h_*a*_*érh*_*3*_*ie/o*- ‘plow’ [18:434]; *h_*2*_*érh*_*3*_*ye/o*- ‘plough’ [10]): Gk. ἀρόω ‘plow, plant’ < PGk. *aroi̯e/o-; Lith. *árti* (*ariù*), Latv. *ar̂t* ‘plow’ < PB *arʔi̯a-; OCS *orati*, Ru. dial. *orát’* (*orjú*), SCr. *òrati* < PSl. *orje/o-; Go. *arjan*, ON *erja*, OE *erian*, OHG *erien* ‘plow’ < PGm. *arjan-; Lat. *arāre* ‘plow’ < PIt. *araje/o-; MIr. *airim* ‘plow’ < PC *ari̯o-

Several European branches attest to a verbal formation *h_*2*_*erh*_*3*_-*ie*- ‘plow’. No direct counterpart of this verb is found in Indo-Iranian. This branch does, however, have a clear manifestation of the derived, widely distributed heteroclitic, *h_*2*_*érh*_*3*_-*ur*, gen. *h_*2*_*rh*_*2*_-*uén*-*s*, cf. Skt. *urvárā*- f. ‘arable land, field’, Av. *uruuarā*- f.pl. ‘(edible?) plant’ < PIIr. *HrHuaraH-, Arm. *harawunk* ‘sowing, seeds, arable land’ < *h_*2*_*erh*_*3*_-*uon*-, Gk. ἄρουρα f. ‘farmland’ < *h_*2*_*erh*_*3*_-*ur*-*h*_*2*_- and OIr. *arbor*, gen. -*e* n. ‘grain’ < PC *aru̯ar, *-*ens* (not with Witczak [19:82] from *H_*2*_*érg*^*w*^*hr̥* [123:196]), proving that the root was present in this branch as well. The instrumental noun *h_*3*_*erh*_*2*_-*tro*- is also found in most core Indo-European branches, Arm. *arawr*, Gk. ἄροτρον, Lith. *árklas*, OCS *ralo*, ON *arðr*, Lat. *arātrum*, OIr. *arathar*, and ToA āre* ‘plow’, ToB *āre* ‘plowing’ has been suggested to continue the same formation through regular loss of the dental [124:386–7, 391].

On the basis of this evidence, it is beyond doubt that a verbal root *h_*2*_*erh*_*3*_- with the meaning ‘plow’ existed directly after the Indo-Anatolian split. This root gave rise to the heteroclitic *h_*2*_*erh*_*3*_-*ur/n*-, present in both Europe and Asia, as well as to the formations *h_*2*_*erh*_*3*_-*ie*- in Europe and *h_*2*_*erh*_*3*_-*tro*- in the European branches and quite possibly Tocharian. Prior to the Indo-Anatolian split, the root *h_*2*_*erh*_*3*_- appears to have had a more primitive meaning. This is suggested by the plausible Anatolian cognate Hitt. *ḫarra*-^*i*^ ‘grind, crush, break up’, which predominantly occurs in non-agricultural contexts [75:8; 125:501]. A vestige of this more primitive meaning is potentially also found in ToB *āre* ‘dust, loose earth’, which lacks a commonly accepted etymology, but may contain the same root PIE *h_*2*_*erh*_*3*_-. It follows from the implied semantic shift that the concept of plowing was likely introduced to the Indo-European family after the dissolution of Indo-Anatolian. Possibly, the root *h_*2*_*erh*_*3*_- had already acquired an association with the crumbling of soil (possibly in connection with hoe agriculture) in early PIE, and therefore was primed for a semantic shift to ‘plow’. Support for such an association potentially comes from Hitt. *ḫārš*-^*i*^ ‘till (the soil)’, which, if not a loan from WSem. *ḥaraš- ‘plow’ [105:III, 185], may be seen as an inner-Anatolian derivation from Hitt. *ḫarra*-^*i*^ [75:312].

Plows were not known during the initial phase of the agricultural expansion, instead appearing as a later innovation [126:415–6]. In the Pontic region, an early antler ard or scratch plow is known from the Maidanetske II–Grebenukiv Yar site dated to the sixth millennium BCE Trypillia BI period [127]. This is the area in which dispersing Indo-European groups could have become acquainted with this tool.

*(H)ieu(H)- (*i̯eu̯o- [44:512]; *i̯éu̯os ~ *i̯éu̯om ‘grain (particularly barley?)’ [18:236]; *yewH_*1*_^*(*^*ó*^*)*^*s* [19:43–4, 54]; *yéwos ‘± grain,? barley,? wheat’ [10]): Hitt. *ewa(n)*- n. ‘type of grain; porridge’ < PAn. *(H)i̯eu̯a-; Skt. *yáva*- m. ‘grain, corn, crop, barley’, YAv. *yauua*- m. ‘grain’, Oss. *jæw* ‘millet’ < PIIr. *(H)i̯au̯(H)a-; Gk. ζειαί f.pl. ‘one-sided wheat, spelt’ < PGk. *i̯eu̯i̯a-; Lith. *javaĩ* m.pl. ‘corn, grain’ < PB *jav(ʔ)a-

Anatolian, Indo-Iranian and Baltic share a common formation *(H)ieu(H)-*o*-. Hittite also shows an *n*-stem inflection, which may be old. Gk. ζειαί additionally presupposes a (collective?) formation *(H)ieu(H)-*ieh*_*2*_-. The previously included ToB *yap* < PTo. *yəp- [18:236; 19:43–4; 21:54–5; 114:371; 121:139–40], with its labial plosive, cannot directly continue the PIE form, however, and appears to be an Indo-Iranian loan [56:246], even if the vowel substitution is unparalleled. The appurtenance of Arm. *ǰov* ‘sprout, branch; dial. string’ [128; 129:138] also remains uncertain, as the semantic shift from ‘grain’ to ‘sprout, branch’ is not transparent.

The deeper etymology of the formation is unclear, partially as the result of difficulties concerning the presence or absence of laryngeals at the beginning [130; 131] and at the end of the root [55:442; 85:147–8]. One suggestion has been to derive it from a verbal root *Hieu(H)- ‘mature’ [18:236–7; 19:43–4; 21:55], cf. ToB *yu*- ‘ripen, mature’ [47:291], but if this root is based on that of *h_*2*_*ei*-*u*- ‘(old) age’, the loss of the laryngeal would be irregular in Hittite. Another proposed connection is with the root *HieuH- ‘graze’, cf. Skt. *yávasa*- n. ‘grass, fodder, pasturage’, YAv. *yauuaŋha*- n. ‘pasture’ < *HieuH-*es*-*o*-, and possibly Kal. *žu*- ‘eat’, Wakh. *yaw*- ‘id.’ [132:555; 133:10507]. However, if this root is present in Gk. εἱαμενή ‘riverside pasture, flood plain, meadow’, continuing *Hieuh_*2*_-*men*-*eh*_*2*_- [134], it is formally incompatible with both Hitt. *ewa(n)*- and Gk. ζειαί.

*Hoket-(i)eh_**2**_**-** (*ok̂etā ‘Egge, Gerät mit Spitzen’ [44:18–22]; *h_*1/4*_*okéteh*_*a*_- ‘harrow, rake’ [18:434–5]; *h_*1/4*_*ek*- ‘rake, harrow’ [135:176]; *h_*3*_*ekéteh*_*a*_- ‘harrow’ [10]): Oss. I *adæg* ‘harrow’ <? PIr. *ātakā-; Lith. *akė́čios*, dial. *ekė́čios* f.pl. ‘harrow’, Latv. *ecê(k)šas* f.pl. ‘harrow’, OPru. EV *aketes* ‘harrow’ < *akētiā- (whence possibly Fi. *äes* ‘harrow’ [136:146; 137:147 fn. 33]); OHG *egida*, OS gl. *egitha*, OE *egeðe* f. ‘harrow’ < *age/iþ(j)ō-; Lat. *occa* f. ‘harrow(?), rake(?), hayrack’, Ital. dial. (Triento) *oca* ‘harrow’ < *otVkā-(?); OW *ocet*, Corn. *ocet*, MBret. *oguet* ‘harrow’ < PC *oke/itā

A word for ‘harrow’ is found in several European branches as well as in Ossetic. No cognates are known from Anatolian or Tocharian. Hitt. *akkala*- ‘furrow’, if at all related [138:26], would show a different formation. The connection of PSl. *esetь, cf. Ukr. *oset’* ‘place for the drying of sheaves’, Biel. *asec’* ‘drying barn’, Pol. dial. *jesieć*, *osieć* ‘grain sieve’ [85:145; 139] is formally and semantically unattractive.

Formally, the Celtic and Italic forms can be combined into *oke/itā under the assumption that Lat. *occa* underwent metathesis prior to the syncope of the medial vowel and assimilation of *tk > *kk* [140:230]. The Germanic form, usually reconstructed as *agiþō-, can be derived from *okitā- as well, but since *ageþjō- is an alternative reconstruction, it may be closer to the Baltic comparandum, Lith. *akė́čios*, *ekė́čios*. Related verbal formations are found in both Germanic and Baltic, viz. OS *gi*-*eggian*, OHG *ecken* ‘harrow’ < *agjan- and Lith. *akė́ti*, *ekė́ti*, Latv. *ecêt* ‘harrow’, but these are not necessarily old and may be back-formations [44:18–22]. Finally, Oss. *adæg* ‘harrow’ can be derived from Proto-(Indo-)Iranian *ātakā- [88:I, 28; 141:197], ostensibly metathesized from *Hoketā prior to the Proto-Indo-Iranian palatalization of the velars.

The distribution of the word presents a dilemma. Given that, within Indo-Iranian, the word is isolated to Ossetic, a prehistoric loan from a European source is possible, e.g. from early Slavic, which is the source of other borrowings related to agriculture [142; 143]. Iron Age steppe Iranians may have acquired knowledge of agricultural practices from neighboring Slavic-speaking groups. Neither *oteka nor metathesized *oketa is attested in Slavic, however. Alternatively, the word would have to be a retention from the core Indo-European stage. Except for the -*ė*- of the Lithuanian form, which could be attributed to influence from the verb [55:10], or from other formations in -*ė́čios*, cf. *vežė́čios* ‘one-horse cart’, there are no clear formal irregularities that would indicate a prehistoric loan; metathesis is hardly an indicator of borrowing. It is therefore possible that some Indo-European groups became acquainted with this implement prior to the final fragmentation of the core Indo-European dialect continuum.

*^(^**ḱ**^)^**eh**_**2**_**p-o/eh**_**2**_**-** (*kāp-, *kəp- ‘Stück Land, Grundstück’ [44:529]; *k̂āpos ~ k̂āpéha (or *k̂eh_*a*_*pos* ~ *k̂eh_*a*_*péh*_*a*_) ‘piece of land, garden’ [18:200]; *kāpos ‘field’ [10]):? Shu. *sɛ̄pc*, Rosh. *sēpc* ‘cultivated field’ < PIIr. *ćāpa-; Gk. κῆπος, Dor. κᾶπος m. ‘plot of land, garden, plantation; (Cypr.) uncultivated piece of land’ < PGk. *kāpo-; Alb. *kopsht*, *kopësht* m. ‘garden; orchard; piece of land granted to a single family’ <? PAlb. *kāp-eśta-; OHG *huoba* f. ‘plot of land, settlement, farmstead’, OS *hōƀa*, MDu. *hoeve* f. ‘hide of land, farmstead’ < PGm. *hōbō-

A formation *^(^*ḱ*^*)*^*eh*_*2*_*p*-*o/eh*_*2*_- can be reconstructed on the basis of Germanic, Greek and Albanian evidence. In the latter language, it appears that an element *kāp- was present from Proto-Albanian, either as an inherited word or as an early Greek loan [63:222], to which a suffix *-*eśta*- was added (cf. *vresht* m. ‘vinyard’ < PAlb. *wain-*eśta*-). Except for in Cypriote, a rather consistent semantic range is observed: in both Germanic and Albanian there is a notion of a plot of land that is sufficient to sustain a household, i.e. a *hide of land*. No further *comparanda* exist in the European languages. The proposed cognate OCS *kapь* f. ‘idol, image’ [144:184] is semantically distant and likely a Turkic loan, cf. Chuv. *kap* ‘size, appearance, form’.

Outside Europe, an important question is whether some Iranian lookalikes, viz. Shu. *sɛ̄pc* and Rosh. *sēpc* ‘cultivated field’, are related. If so, the root would have to be reconstructed with a palatovelar and the Albanian form explained as a loan from Greek [18:8, 200]. In isolation, these East Iranian forms indeed allow for such an interpretation. Parallel to Shu. *zimc* ‘field’ < *j́^*h*^*ami*-*čī*-, the productive suffix *-*čī*- appears to have been added to a base *ćāpa- [145:74], after which it caused umlaut. Within the wider Iranian context, however, this *ćāpa- is not necessarily isolated. It may have a more immediate cognate in Psht. *sābə́* m.pl. ‘greens, vegetables; a fodder grass’ [81:73]. This form has previously been derived from *ćapa- [104:283], but since PIr. *ă in open syllables yields Psht. *a* or *Ø* depending on the accent [146:176], a reconstruction with *ā is more attractive. A variant with *a does seem to be present in MP *sbz*, *spz*, NP *sabz* ‘green, fresh’. This adjective has been interpreted as continuing *ćapačya- or *ćapači(H)a-, possibly created to a formation *ćapaka- for which Bact. *σαβαγο* ‘crop’ < *ćā̆pā̆kā̆- may be compared [147:261]. More probably, the adjective *sabz*, which itself served as the base for the inner-Persian derivation *sabzī* ‘greenness, verdure, vegetable’, started out as a noun, continuing *ćapačī- ‘vegetation’. Since the Iranian variant with *ă is formally incompatible with the root of *^(^*ḱ*^*)*^*eh*_*2*_*p*-*o/eh*_*2*_-, it is possible that all the Iranian forms are unrelated. Instead, they may rather be cognate with Skt. *śāpa*- ‘flotsam’ and Lith. *šãpas* m. ‘straw’, pl. ‘flotsam’ < *ḱop-*o*- [77:II, 629; 144].

†*keres- (*k̂er-*2*, *k̂erə*-, *k̂rē*- [44:577]; *kers [19:82]; *keres- ‘millet’ [10]): Hitt. *karaš* n. ‘wheat, emmer-wheat’; Kal. *káras*, *karazí* ‘a kind of grain like millet or bajari’; ON *hirsi* m. ‘millet’, OHG *hirsi*, *hirso* ‘millet’ < PGm. *hersja-

A formation *keres- ‘millet’ is given by Mallory [10], based on Hitt. *karaš*, PGm. *hersja- and Kalasha *káras* (and similar forms in Dardic and Nuristani). This reconstruction, which resembles an *s*-stem, is untenable for multiple reasons. First of all, neuter *s*-stems lose their final syllables in Kalasha, cf. *me* ‘fat’ < Skt. *médas*- ‘fat, marrow’, *sar* ‘lake < Skt. *sáras*- ‘lake, pool’, meaning that the attested *káras* cannot reflect PIIr. *kar(H)-*as*- in underived form. More fatally, PIE *ker(H)-*es*- should according to the known sound changes have resulted in PIIr. form *čar(H)as- rather than *kar(H)as-.

The comparison can be partially saved by reconstructing *ḱer(H)-*s*- (or *kers- [19:82]), a protoform that works for Anatolian and Germanic (but not for Indo-Iranian). This would then be an *s*-stem created to the PIE root *ḱerH- ‘feed’, cf. Gk. κορέννυμι ‘satiate, fill’ < *ḱorh_*1*_-(?), Lat. *Cerēs*, -*eris* f. ‘goddess of grain and fruits’ < *ḱerH-*es*-, Lith. *šérti* ‘feed (animals)’ < *ḱerH-, Alb. *thjer* m. ‘acorn’ < *ḱerH-*o(s)*- [18:248–9]. However, Hitt. *karaš* has alternatively been linked to *ǵ^**h**^**ersd-** (q.v.). In addition, the association of PGm. *hersja- with millet (*Panicum miliaceum*) is probably secondary, given the absence of this crop in South Scandinavia prior to the 2nd millennium BCE [148:146]. In view of the semantics of Lith. *šérti* and Alb. *thjer*, it is likely that this meaning developed from *‘(animal) feed, mast’.

In conclusion, no word for millet can be reconstructed for Proto-Indo-European on the basis of the aforementioned forms.

?*meiǵ^**h**^**-** (*meiĝ(h)- ‘barley’ [18:51]; *meiĝ^*h*^- ‘± grain’) [10]: Khot. *mäṣṣa*-, *miṣṣa*- ‘field for seed’ (whence ToA *miṣi*, ToB *miṣe* ‘field’ [56:268–9]) < PIIr. *mixša-(?); Lith. *miẽžiai* m.pl. ‘barley’, Latv. *mìeži* m.pl. ‘barley’, OPru. *moasis* ‘barley’ < *maižia-; Latv. *màize* f. ‘bread’ < *maižiā-;? OIr. *míach* n./f. ‘a grain measure, bushel’ < PC *meiko(s)- or *meig-(?)

On the basis of the Baltic forms and Khot. *mäṣṣa*-, *miṣṣa*-, a reconstruction *miǵ-*so*- has previously been proposed for Indo-Iranian [67:333]. However, after the discovery of Winter’s law, it became clear that the intonation rather mandates a root *meiǵ^*h*^- with a voiced aspirate [cf. 55:798–9]. To save the etymology, the protoform has subsequently been modified to *miǵ^*h*^-*so*- [93:129]. Unfortunately, the implied cluster *-*ǵ*^*h*^*s*- > PIr. *j́^*h*^*ž* > *ž does not regularly yield Khot. -*ṣṣ*-, which indicates a voiceless sibilant [149:196–8]. Consequently, the etymology cannot be maintained.

Alternatively, Khot. *mäṣṣa*- can be derived from PIE *miḱ-*so*-, and then connected to OIr. *míach* ‘a grain measure, bushel’, assuming that the latter continues PIE *meiḱ-*o(s)*- [93:129]. Though technically possible, the comparison has been called “extremely doubtful” [150:215 fn. 4]. The alternative suggestion that OIr. *míach* acquired its *k by 1) devoicing before *s* in a nominative *meiǵ^*h*^-*s*, and 2) subsequent leveling to the other cases, is not much better, as the analogy is without parallels [151:126].

Within Iranian, Sogd. M *myj’* ‘lens, lentil’ has additionally been adduced to further substantiate an *s*-stem *miǵ^*h*^-*so*- [93:129]. Since there is no other Iranian evidence for such an *s*-stem, however, a more straightforward protoform would be *maij́^*h*^*iākā*-, perhaps for older *maij́^*h*^*iukā*-, which would bring it closer to the Baltic attestations continuing *moiǵ^*h*^-*io*-. However, *myj’* is a *hapax legomenon* whose meaning is difficult to establish. It occurs exclusively in a cosmological context and the translation as ‘lens’ [152:316] appears at least partially inspired by the etymological identification with MP *mycwk*, *myšwk*, NP *mīžū* ‘lentil’. Yet the Persian form resists derivation from *maij́^*h*^*iukā*-: since *ī* cannot continue PIr. *ai and *ž* cannot regularly reflect *ǵ^*h*^*s* or *ǵi̯, it can only be maintained as a loan from unattested Sogd. *myjwk(h) (cf. NP *rēž* ‘desire’ ≪ Sogd. *rēž* ‘desire, lust’) or from a corresponding form from another Iranian language in which *j́^*(h)*^*i̯* > *ž. In conclusion, this etymology is plagued by many formal and philological uncertainties. While difficult to completely reject on formal grounds, the comparison remains doubtful.

Finally, Khot. *biṃmīysā* has been compared, under the assumption that it continues a compound with Khot. *bījä* ‘seed’, i.e. *bāi-*maizākā̆*- [67:285] or *bija-*miysā*- “grain plant” [93:129]. However, the assumed loss of *j appears to be *ad hoc* and since the origin of *biṃ*- remains unclear, the analysis of *biṃmīysā* as a compound cannot be substantiated.

†*pano- (*pank-, *pang- ‘Büschel der Hirse’ [44:789];? *pano- ~ *paniko/eh_*a*_- ‘millet’ [18:383]; *pano- [10; 135:65]).

This etymology is based on the comparison of Lat. *pānicum* ‘millet’ and various Iranian forms, including Shu. *pīnǰ*. The resemblance is superficial, however. Within Italic, Lat. *pānicum* is evidently derived from Lat. *pānus* m. ‘tuft, tufty grass, ear of millet’ [44:789], with a velar suffix that is found in *triticum* ‘wheat’ as well as in *alica* and *milica (cf. Ital. *melica*, OProv. *melga* ‘sorghum’). The Iranian forms are Indic loans, ultimately going back to Skt. *priyáṅgu*- ‘foxtail millet (*Setaria italica*)’ [104:284; 145:57–8].

*peis- (*peis- ‘remove the hulls from grain, grind, thresh’ [18:581]; *peis- ‘grind, thresh’ [135:167]; *peis- ‘grind’) [10]:? Hitt. *peš(š)*-^*zi*^ ‘rub, scrub’ < PAn. *pe(i)s-; ToA *psäl*, ToB *pīsäl* ‘chaff (of grain), husk’ < PTo. *p’əs-l-; Skt. *peṣ* ‘crush, grind’, YAv. *pišaṇt*- ‘crushing, bruising’ < PIIr. *paiš-; Gk. πτίσσω ‘grind, winnow’ < PGk. *pis-i̯e/o-; Lith. *paisýti* ‘beat (off) chaff from grain’, Latv. *pàisît* ‘pound or break flax’ < PB *pais-ī/ā-; Ru. *pšenó* n. ‘millet’, Sln. *pšénọ* n. ‘peeled grain, millet’ < PSl. *pьšeno; OHG *fesa* f. ‘chaff’ < PGm. *fisōn-; Lat. *pīnsō* ‘crush, pound’ < PIt. *pins-e/o-

A root *peis- is widely attested in the Indo-European languages, with meanings suggestive of an association with cereal processing, specifically the dehusking of grains by grinding, cf. derivations such as Lith. *piẽstas* m. ‘(wooden) mortar, pestle’, Ru. *pest* m. ‘pestle’ < PBSl. *paista- and MDu. *visel*, Du. *vijzel* c. ‘mortar, pestle’ < PGm. *fīsila-. We may further connect ToA *psäl*, ToB *pīsäl* ‘chaff’, which has previously been connected to a verbal base *pes- ‘blow’ [59:417]. As a consequence, the element *peis- must be admitted to the oldest stratum of core Indo-European. This suggests that the corresponding language community may have been familiar with the technique of dehusking cereals by grinding them with mortars and pestles. Pestles are well known from Yamnaya burials [3:309; 153:240]. However, these tools were multifunctional and could have been used to process wild (grass) seeds or to crush salt or ochre. As such, they are not exclusive indicators of agriculture. Nevertheless, the linguistic association with cereal processing is highly pervasive and suggests that they were used for this purpose by the majority of the core Indo-European subgroups.

In the absence of a straightforward cognate in Anatolian, it is not known whether the root *peis- occurred in Indo-Anatolian and with what semantic range. It has been suggested that Hitt. *peš(š)*-^*zi*^ ‘rub, scrub’ is derived from the same root [75:669], in which case Anatolian would attest to a more primary semantic stage. However, the latter has alternatively been connected to Skt. *psā́ti* ‘chew, devour’ and Gk. ψάω ‘rub, grate, stroke’ < PIE *b^*h*^*esH*- [cf. 82:98].

*pelH-ou- (*pelṓus, *pelu̯-*ós* [44:802]; *pelo/eh_*a*_- ‘chaff’ [18:104]; *pelo/eh_*2*_- ‘chaff’ [10]): Skt. *palā́va*- m. ‘chaff, husks’ < PIIr. *par(H)āu̯a-; Lith. *pẽlūs* m.pl. ‘chaff’, Latv. *pęlus* f.pl. ‘chaff’, OPru. EV *pelwo* ‘chaff’ < PB *pelʔu(a)ʔ; OCS *plěvy* f.pl. ‘chaff’, Ru. *polóva* f. ‘chaff’, SCr. *pljȅva* f. ‘chaff’ < PSl. *pèlva;? Lat. *pulvis*, -*eris* n. ‘dust, powder’ < PIt. *pe/olou̯-

An amphidynamic *u*-stem *pélH-*ou*-, *plH-*u*-*ós*, can be reconstructed on the basis of Indo-Iranian, Balto-Slavic and possibly Italic. Skt. *palā́va*- appears to be a direct thematization of this *u*-stem (cf. Skt. *áṅgāra*- ‘coal’ < *h_*1*_*e/ong*^*w*^-*ō̆l*-*o*- for a parallel). The Balto-Slavic forms rather point to an extension with a collective suffix *-*h*_*2*_. Lat. *pulvis*, -*eris*, with analogical *-*is*- after *cinis*, -*eris* n. ‘dust’ [46:257], probably continues the same formation, i.e. PIt. *pelVu̯- or *polVu̯-. Alternatively, it can be grouped with ON *fǫl* n. ‘thin layer of snow’ < *falwa- (whence Far. *følva* ‘cover in a thin layer (of snow, butter, flour)’) and Alb. *pall* m. ‘finely milled flour, chaff and dust from harvested grain’ < PAlb. *palwa- < *polH-*uo*-. In addition to these full-grade forms, a zero-grade root variant *plH-*u*- may possibly have served as the base for Gk. παλύνω ‘strew, sprinkle; bestrew, besprinke; smear, cover lightly’.

It is possible to derive the *u*-stem from a root *pelH-, as found in Gk. πάλλω ‘sway, rock’, e.g. through a semantic shift from original ‘shake’ to secondary ‘sieve’ (cf.? *k^**w**^**eh**_**2**_**t-i-**). If the original meaning of this *u*-stem was ‘sprinkling, scattering’, the Greek, Germanic and Italic attestations pointing to ‘dust, powder’ would be conservative compared to those found in Albanian, Balto-Slavic and Indo-Iranian. The evidently agricultural meaning ‘chaff’ appears dominant in Balto-Slavic and Indo-Iranian, a semantic narrowing that possibly constitutes a shared innovation.

To the same root *pelH-, a number of isolated and possibly independent formations can be found. Lat. *palea* f. ‘chaff, dross, straw’ can be taken back to PIt. *palejā- < *plH-*ei*-*eh*_*2*_-, with a collective suffix. Gk. πάλη ‘fine flour, dust’ appears to continue *plH-*eh*_*2*_-. In addition, Alb. *pjalm* m. ‘pollen; flour; dust; fine snow’, previously connected to *pjell* ‘beget, procreate’ [63:323], can alternatively be derived from *pelH-*m*-. No certain cognates are available from Tocharian or Anatolian. A possible continuant of a root *pelh_*2*_- is found in Hitt. (Luw.) *palḫ*-, perhaps ‘shatter, split open’ [105:63–4; 154:P, 63–4], but the attestation of this verb is too weak to allow for a comparison.

*se-sh_**1**_**-io-** (*sasi̯o- ‘Feldfrucht’ [44:880]; *ses(i̯)ó- ‘grain, fruit’ [18:236]; *sᵉsyā, *sᵉsyom [19:41–2]; *ses(i)o- ‘± grain’ [10]): Skt. *sasyá*- n. ‘corn, grain’ < PIIr. *sas(H)i̯a; YAv. *hahiia*- adj. ‘pertaining to grain’ < *sas(H)i̯a-; Skt. *sasá*- n.(?) ‘corn-field, corn’ < *sas(H)a-; W *haidd*, Corn. *hêth*, Bret. *heiz* ‘barley’ < PC *sesi̯o-

A reconstruction *ses-*io*- has been proposed on the basis of Celtic and Indo-Iranian. The alternative reconstructions *sas-*i̯o*- [44:880; 104:23] and *sh_*1*_*s*-*io*- [21:57] appear to be primarily based on the Latin regionalism *asiam* ‘rye’, attributed by Pliny to the Taurini in Northern Italy. The term has been emended to *sasia, so as to compare it to alleged Proto-Celtic *sasi̯o- and some Occitan words including Cat. *xeixa*, Val. *seixa* ‘white wheat’ < PRom. *sassia. However, the reconstruction of Proto-Celtic *a is contradicted by the Welsh vocalism [51:318–9] and the double *ss* implied by the Occitan material can be explained neither from PC *sasi̯ā nor from PRom. *sasia [116:XI, 257].

Through internal reconstruction, the proposed *ses-*io*- can be interpreted as a reduplicated formation to the root *seh_*1*_- ‘sow’, extended with the collective *io*-suffix. If correct, the underlying meaning of the word must have been ‘collections of seeds’. The meaning of the root *seh_*1*_- ‘sow’ itself may have developed in core Indo-European from Indo-Anatolian ‘put in (the ground)’, cf. Hitt. *šai*-^*i*^ ‘impress, prick’ < *sh_*1*_-*oi*- [125:504]. If correct, the creation of the formation *se-*sh*_*1*_-*io*- must likewise postdate this semantic shift.

A formally close formation is Skt. *sasá*- n. ‘herb, grass, grain’ (RV+). It lacks the *io*-suffix and thus presupposes PIIr. *sasa- < PIE *ses-*o*- [155] or *se-*sh*_*1*_-*o*- [156:180]. A formal resemblance exists in Hitt. *šēša*- ‘fruit’ [104:280; 155:26–8]. However, the similarity of the two formations may be deceptive [157:269 fn. 26]. Hitt. *šēša*- does not seem to have contained a laryngeal, in view of the lack of expected geminate -*šš*- < *-*sh*_*1*_-. For this reason, the alternative derivation from the verb *šiš*-*zi* ‘prosper, proliferate’ is preferable [75:756–7]. This verb is usually derived from a root *šišd*- [158:166], and if correct, it may be a reduplicated present cognate with Ved. *sidhyati* ‘succeeds’ < *sHd^*h*^-*ie/o*-.

*srp-o/eh_**2**_**-** (*serp- ‘Sichel, krummer Haken’ [44:911–2]; *sŕ̥po/eh_*a*_- ‘sickle’ [18:8]; *srpo/eh_*2*_- ‘sickle’ [10]): Gk. ἅρπη f. ‘sickle’ < PGk. *sr̥pā-; Latv. *sirpis*, *sìrps* m. ‘sickle’ < PB *sirp(i)a-; SerbCS *srъpъ*, Ru. *serp*, gen. *serpá*, Pol. *sierp*, SCr. *sȓp* m. ‘sickle’ < PSl. *sьrpъ

A thematic formation *srp-*o/eh*_*2*_- can be reconstructed on the basis of Balto-Slavic and Greek attestations. It was evidently derived from the (marginally attested) PIE root *serp- ‘cut, prune’. This root is also found in Lat. *sarp(i)ō* ‘cut off, trim, prune’ < *srp-*ie*-, apparently with regular vocalization of *CRCC- to *CarCC- [46], and in OHG *sarf*, MHG *sarpf*, MDu. *sarp*, Du. obs. *zerp* adj. ‘severe, sharp’ < PGm. *sarpa- (not *sarfa- [*pace* 44:911–2] < *sorp-*nó*- (with Kluge’s law). The resemblance to Akk. *sirpu* ‘shears’ [88:IV, 242] is coincidental, as the root of this formation appears to be metathesized from *spr ‘cut the hair, shave’ [159].

In addition, OIr. *serr* f. ‘sickle’ is often derived from PC *serϕā, which would continue a second, full-grade formation *serp-*eh*_*2*_-. The change PC *rϕ > *rr* is unconfirmed, however [160:154b; 161:389], and the Old Irish word can alternatively be derived from *sersā, potentially cognate with or even borrowed from Lat. *serra* f. ‘saw’ < *sers-*eh*_*2*_-, cf. Lat. *sar(r)iō* ‘hoe, weed’. Still, the possibility that *serr* continues an independent formation to the root *serp- cannot be rejected and finds a parallel in the derivation of OFr. *sarpe* f. ‘pruning knife’, Fr. *serpe* f. ‘sickle; billhook’ from OFr. *sarper* ‘cut off’ [116:XI, 234].

Some additional Indo-Iranian comparanda occurring in the literature are highly problematic. The appurtenance of Skt. (lex.) *sr̥pa*-, *sr̥prá*- m. ‘moon’ is based on the conjecture that its meaning developed through “sickle-shaped moon”. Oss. I *xsyrf*, D *æxsirf* ‘sickle’ was not inherited from Proto-Indo-Iranian *srp-*a*-, but rather borrowed from Slavic [88:IV, 242; 142:8–9]. Finally, the frequently compared Skt. *sr̥ṇī́*-, *sŕ̥ṇī*- f. ‘sickle’ < *sr-*niH*- cannot be accepted as a cognate since it contains no reflex of *p.

In conclusion, it is possible to postulate a dialectal Indo-European, i.e. European, formation *srp-*o/eh*_*2*_-, meaning ‘sickle’. Remains of sickles and reaping knives are not known from Yamnaya contexts except for five late sites in the West Pontic [162:48] (see also Fig 3). As a result, it is possible to conclude that Indo-European speakers originally did not have a word for ‘sickle’ (or ‘reaping knife’), but that a subset of them created one after their departure from the homeland.

*uers- (*u̯ers- ‘am Boden schleifen’ [44:1169–70]; *u̯ers- ‘thresh’ [18:8, 581]; *wers- ‘thresh’ [10]): Hitt. *u̯arš*-^*i*^ ‘sweep, wipe; pluck, harvest’ < PAn. *u̯a/ors-; ON *vǫrr* m. ‘pull of the oar’ < PGm. *warzu-; Lat. *verrō*, -*ere* ‘scrape, sweep, brush’ < PIt. *wers-; Latv. *vā̀rsms* m. ‘layer of grain (spread out for threshing)’ < PEB *varsma-; RuCS *vresti* (*vьrxu*) ‘thresh, SCr. *vrijȇći* (*vŕšem*) ‘thresh’ < PSl. *versti (vьrxǫ); RuCS *vrachъ* m. ‘threshing’, Ru. *vóroch* m. ‘pile of grain’ < PSl. *vorxъ

The root *uers- is attested in Anatolian and several European branches. The original meaning was probably ‘sweep, wipe’ [44:1169–70], which is attested in multiple branches. In Anatolian, the verb occurs in contexts associated with cereal processing, i.e. harvesting and wiping the threshing floor, but in view of the lack of these meanings in Germanic, these may be secondary developments from more general ‘wipe’. In Balto-Slavic, too, the root appears to be applied to the wiping of threshing floors, where harvested grain was laid out for tramping. This semantic narrowing could be old in view of the Hittite cognate [163], but with this method of threshing it is easy to see how it alternatively could have occurred independently in the branches involved, especially where the original meaning ‘wipe’ is retained as well. A potentially stronger candidate for a core Indo-European verbal syntagm *pers-*ons g*^*wh*^*en*-*ti* ‘thresh sheaves’ has been postulated by Wachter [164].

### 3.2. Additional Indo-European terms proposed elsewhere

?*ǵ^**h**^rud-o- (*ghrū̆dom [19:119]): Lith. *grū́das*, Latv. *grûds* m. ‘grain’ < PB *gruʔda-; OE *grotan* m.pl. ‘hulled and crushed grain’, E *groats* ‘groats’, WFri. *grôt* ‘(pearl) barley’ < PGm. *gruta(n)-

A form *g^*h*^*rud*-*o*- has been proposed on the basis of Germanic and East Baltic forms [19:119]. However, it cannot be excluded that these are independent derivations from PGm. *greutan- ‘grind’, cf. OHG *for*-griozan*, MHG *griezen* ‘crush, grind’ and Lith. *grū́sti* (*grū́džiu*, *grū́du*) ‘thrust, pestle, stamp’, Latv. *grûst* ‘stamp, press’, respectively, both continuants of a verbal root *ǵ^*h*^*reud*- ‘crush’. Within Germanic, the parallel OHG *gruzzi* n. ‘grits’, G *Grütze* f.pl. ‘groats’, MDu. *gorte* n. ‘porridge’, Du. *gort* c. ‘pearl barley, groats’ < *grutja/ō- may be compared. The same root *ǵ^*h*^*reud*- has also been proposed as the derivational base of ToA *oṅkriṃ*, B *oṅkarño*, *onkorño* ‘porridge, rice gruel’ through *h_*1*_*n*-*ǵ*^*h*^*rud*-*n*-*i(H)o*- [165:170–1; 166:137–8], but other interpretations cannot easily be excluded. The inclusion of Alb. *grurë*, Gheg *grunë* f. ‘wheat’, as if from *ǵ^*h*^*rud*-*(i)neh*_*2*_- [20], cannot be accepted, as a nasal resulting from *-*dn*- does not rhotacize. The alternative derivation from *ǵrH-*u*-*no*- [69:278–9] finds no support outside Albanian.

?*h_**2/3**_**elǵ**^**(h)**^**-** (?*h_*2/3*_*elǵ(h)*- ‘grain or millet?’ [18:237];? *h_*2/3*_*elǵ(h)*- [135:164]): Hitt. *ḫalki*- c. ‘barley, grain’ < PAn. *h_**2**_**elKi-**; MP *ʾrzn*, P *arzan* ‘millet’ (< Parth.), Sogd. *ʾrzn* ‘id.’, Psht. *ğdən* ‘id.’, Yd. *yurzun*, Wakh. *yirzn* < PIIr. *Harj́^**(h)**^**ana-**(?)

Hittite *ḫalki*- appears isolated, and can be derived from PIE *h_*2*_*el(H)K*-*i*- [75:274–5; 167:54]. This root can technically be compared to a cluster of Iranian terms pointing to PIIr. *Harj́^*(h)*^*ana*- [18:237; 81:29]. However, due to the formal ambiguities of both the Hittite and the Iranian forms, the comparison is impossible to substantiate. Other comparanda can be rejected out of hand. Gk. ἄλιξ m. ‘groats of einkorn or rice wheat’ cannot be regularly related to either the Anatolian or the Iranian forms. If not a loan from a foreign source [168:69], it may, in view of the meaning ‘groats’, have been derived from the verb ἀλέω ‘grind’ < PIE *h_*2*_*elh*_*1*_-, with a suffix -ικ [44:28–9]. The Greek word in turn appears to have been the source of Lat. *alica* f. ‘spelt, spelt grits’ and its (slightly divergent) Romance continuants, Sp. *álaga* ‘a type of wheat’ (< PRom. *alaca), Rom. *alác* ‘spelt, einkorn wheat’ (< *allacus), which thus add no new information. The occasionally compared ToB *lyekśiye* ‘millet’ is certainly unrelated and remains etymologically obscure [56:245].

*h_**2**_**eǵ-ro-** (*aĝ-*ro*-*s* ‘Feld, Flur’ [44:4–6; 79:8]; *h_*a*_*eĝros* ‘field, pasture’ [18:200]): Skt. *ájra*- m. ‘plain’ < PIIr. *Haj́ra-; Gk. ἀγρός m. ‘field, land, countryside’; Go. *akrs*, ON *akr*, OE *æcer*, OHG *ackar* m. ‘cultivated field’ < PGm. *akra-; Lat. *ager* m. ‘field, farm, terrain’ < PIt. *agro-

A formation *h_*2*_*eǵ*-*ro*- can be reconstructed on the basis of the European centum branches and Indo-Iranian. The original meaning was probably ‘field’, i.e. one on which cattle can be driven, in view of the transparent derivation from the PIE root *h_*2*_*ég*- ‘drive’. In the European languages, most notably Germanic, the word became associated with a cultivated field. This semantic shift is evidently late, however, as the less derived meaning still also persists in Italic and Greek.

†*h_**2**_**eui(ḱ/ǵ**^**h**^**)s-** (*au̯iĝ- ‘Grasart, Hafer’ [44:88]; *h_*a*_*eu̯isos* [18:7, 409]; *H_*2*_*awiĝ*-*i*- [19:66]; *h_*a*_*ewis* [135:166]):? Yazg. *wis*, Taj. Wj. *gis* ‘oats’ < PIIr.? *(H)(a)uić-; Lith. *aviža* f. ‘id.’, Latv. *àuza* f. ‘id.’ < PEB *avižaʔ-; OPru. *wyse* ‘oats’ < PWB *vižiā̆-; Ru. *ovës* ‘id.’, SCr. *òvas* ‘id.’ < PSl. *ovьsъ; Lat. *avēna* ‘oats’ < PIt. *awe(C)snā-

A similar word for oats occurs in several European branches, but their unification into an IE protoform is problematic. Lat. *avēna* has been lumped with PEB *avižaʔ- and PSl. *ovьsъ under a PIt. protoform *aweKsnā-, but the vocalism does not match and the Baltic and Slavic forms themselves cannot be reconciled with each other. In addition, OPru. *wyse* appears to continue PWB *vižiā̆-, without the initial vowel that is observed in the other forms. Given these irregularities, no single reconstruction can be offered, suggesting the possibility of a prehistoric loanword [169:100]. Rather than projecting the Balto-Slavic and Italic protoforms back into PIE, i.e. as *h_*2*_*euiḱ*-, *h_*2*_*euiǵ*^*h*^- and *h_*2*_*eue(K)s*-, a root-final “spirant of indeterminate voicing would account for the Italic and Balto-Slavic forms more concisely” [170:404]. Thus, the pre-forms of the various branches can be reconstructed with affricates, viz. *(a)widz- for Baltic, *awits- for Slavic and *awe(t)s- for Italic. The unstable initial vowel is reminiscent of the *a*-prefix identified in a number of Pre-Indo-European loans [47:294–5; 171; 172:518].

Outside Europe, a few other forms have been adduced. The connection of ToB *ysāre* ‘wheat’ [173:396] seems unwarranted [56:251–2], but Khot. *ha̮u* ‘a type of grain’ can be derived from PIIr. *Hau(V)ć- or *Hau(V)j́- [67:497], despite other proposals [80:95; 93:220], and Yazg. *wis*, Taj. Wj. *gis* ‘oats’ could possibly continue PIIr. *(H)(a)uić- [20:220]. Given the eroded character of these words, it is difficult to reject a connection to the European cluster [104:282]. However, since the European comparanda are irregular, such a connection can only be maintained through the assumption of an early *Wanderwort*. In such a scenario, we could potentially also mention an irregular West Uralic word for ‘wheat, spelt’: Fi. *vehnä*, Mrd. *viš* < *wešnä vs Ma. *wištə* < *wäšnä [cf. 174:157].

The earliest evidence for cultivated oats is found across Germany from the LBA [175]. Domesticated oats may have spread from the west to the east along a steppe route [176:68] and it is possible that (Indo-)Iranian speakers participated in this process. Interestingly, the Iranian protoform *Hauić- has its closest match in Pre-PSl. *awiś-.

?*kok-ro- ~ *kork-io- (*korkri̯o- [44:529]): OGutn. *hagri* m. ‘oats’ < PGm.? *hagran-; OIr. *corca*, *coirce* m. ‘oats’, MW *keirch* ‘oats’ < PC *korki̯o-

A term referring to ‘oats’ is found in Germanic and Celtic. If not a loan from one branch to the other, parallel borrowing from a third source is conceivable. This might account for the alternation between *-rk- and *-kr-, but it is also possible that an inherited protoform *kork-*ro*- was dissimilated by the two branches independently into *kok-*ro*- and *kork-*o*-, respectively.

Strikingly, both the Germanic and Celtic forms may originally (also) have meant ‘hair’. North Germanic *hagran- appears to be derived from *hagra-, cf. Nw. dial. *hagr*, *harg* ‘horse hair’, although it is clear that the meaning ‘oats’ must have arisen early in view of the Finnic loan *kakra ‘oats’, cf. Fi. *kaura*, Est. *kaer*, Liv. *kaggõrz*. The derived *ja*-stem *hagrja-, cf. Da. *hejre* c. ‘bromegrass’, if not simply a collective, can be analyzed as “oats-like”. In Celtic, OIr. *coirce* appears identical to OIr. *coirce* m. ‘crest, tuft’, a formation derived from *corc* ‘hair’ < PC *kork-*o*- [17:594]. The occasionally adduced Alb. *thekër* f. ‘rye’ < PAlb. *ϑakri-(?) is almost certainly unrelated and appears to have been derived, within Albanian, from *thek* m., *thekë* f., dial. *thak* f. ‘awn, tassel, fringe’ [177:91 ff.].

?*k^**w**^**els-** (*k^*w*^*el*-*1*, *k^*w*^*elə*- [44:639–40]):? Hitt. *gulš*-^*zi*^ ‘carve, engrave’; Skt. *karṣ* ‘pull, drag; plow’, YAv. *karšaiti* ‘draw; plow, sow’ < PIIr. *karš-;? Gk. τέλσον n. ‘end of the field, where the plow is turned’ < PGk. *k^**w**^**els-o-**(?)

A root *k^*w*^*els*- in the meaning ‘make furrows’ has been reconstructed for Proto-Indo-European based on material from Anatolian, Indo-Iranian and Greek [cf. 82:338–9]. The meaning of the Hittite verb has been explained as secondary from ‘make furrows [75:492–3], but given the basal character of Anatolian it seems more attractive to see the meaning ‘carve’ as primary, and ‘make furrows’ as secondary. However, the phonetic reading of the Hittite verb as *gulš*-^*zi*^ is challenged by Waal [178], who argues that it rather must be taken as a sumerogram. If correct, this GUL-*š*-^*zi*^ can no longer be regarded as a continuant of an inherited root *k^*w*^*els*-. As a result, the root *k^*w*^*els*- is demoted to the core Indo-European stage. Since Gk. τέλσον, however, has two rivaling etymologies, one taking it from the root *k^*w*^*els*- [168:1464], the other from τέλος ‘end’ [179:260 f.], its appurtenance is uncertain as well. It cannot therefore be excluded that the meaning ‘make furrows’ that is found in Indo-Iranian with the root *karš- developed from ‘drag’ within this branch [180:484]. This may have happened under the influence of the semantically close *karH- ‘sow’ [181:241–3], with which it is suppletive in part of Iranian. Such a scenario is indeed supported by the fact that these two meanings are still found side by side in both Indic and Iranian.

†*proḱ-so- (?*prok̂om [19:81]): ToB *proksa* pl. ‘?’; OPru. *prassan* ‘millet’ < PWB *pra^**(**^**ś**^**)**^**a-** (if not < Sl.); OCS *proso*, Ru. *próso*, SCr. *prȍso* n. ‘millet’ < PSl. *proso

The Tocharian and Balto-Slavic forms have been connected through a protoform *proḱ-*so*- [59:454; 182:196–7]. However, the meaning of this Tocharian hapax is uncertain and *proḱ-*so*- would rather develop into **prekse [56:259–60]. The alternative connection of the Balto-Slavic forms with PGm. *hab(e/a)ran- and Jass. *zabar*, by metathesis from *ḱop-*ro*- [19:81], is formally and semantically challenging.

*puH-ro- (*pū-*ro*- ‘Korn(frucht)’ [44:850]; *puH_*x*_*rós* [18:639]; *pūrós [19:190]): Gk. πῡρός, pl. πῡροί m. ‘wheat’ (whence Ge. *ṗuri* ‘bread’ [183:190]) < PGk. *pūro-; Lith. *pūraĩ* m.pl. ‘winter wheat’, Latv. *pùr̨i* m.pl. ‘winter wheat’ < PB *puʔra-; CS *pyro* n. ‘spelt’, SCr. *pȉr* m. ‘spelt’ < PSl. *pyro, *pyrъ

A European formation *puH-*ro*-, referring to a cereal, can be reconstructed on the basis of Balto-Slavic and Greek. The occasionally adduced OE *fyrse* ‘furze’ < PGm. *fursjō- is formally and semantically too distant to be considered a reliable cognate. Outside Europe, Skt. *pūra*- ‘sort of cake’ has traditionally been compared [19:94; 44:850; 66:94], but it is better considered unrelated [77:III, 332]. As a result, the formation cannot be given core Indo-European status.

The transmission of the Baltic word raises some questions. Due to its confinement to Samogitian dialects, it is considered a Curonianism, which may explain the consistently circumflex accent. The isolated East Latvian form *pûri* has been adduced to secure the original acute [184:71]. Lith. dial. *pū̃rės* f.pl. ‘cottongrass’ and OPru. EV *pure* ‘bromegrass’ appear to continue PB *puʔriaʔ-, likely derived from the same base *puʔra-. A similarly secondary formation *pyrьjь, derived with a suffix *-iHo-, exists in Slavic, cf. Ru. *pyréj*, Pol. *perz* m. ‘couch grass’.

It is possible that *puH-*ro*- was derived from an inherited element *peuH-. Since it is formally identical to the Italo-Celtic adjective *puH-*ro*- (Lat. *pūrus* ‘clean, pure’, OIr. *úr* ‘fresh’, W *ir* ‘fresh, green’), it may be a nominalization thereof, with an original meaning “pure wheat”, i.e. a free-threshing wheat that can be winnowed [123:198–9; 185:38–42]. More plausibly, the cereal term was derived directly from the root *peuH- ‘clean’, cf. Skt. *pav*^*i*^ ‘become clean’, after it had become associated with the cleaning of cereals, i.e. with winnowing in (a subsection) of the core Indo-European branches. If correct, we must assume that the semantic shift occurred in the branches in which we find the cereal term *puH-*ro*-, viz. Greek and Balto-Slavic. The semantic shift is directly attested, albeit rather marginally, in Vedic, in the collocation in RV X.71.2a *sáktum iva títaünā punántaḥ* ‘purifying [her] like coarse grain by a sieve’ [186:1491] and in the derivation *pávana*- ‘sieve, winnowing basket’. Although less certain, we may further adduce Alb. *pah* m. ‘flour, chaff, dust’ < *pouH-*sk*-*o*- (not *pou-*io*- [187:47]), possibly derived from a secondary *sk*-present *puH-*sk*-*e/o*-. In the West European centum languages, Germanic attests a causative(-iterative?) formation, i.e. OHG *fewen*, *fouwen* ‘sift (ashes, dust)’ < *pouH-*eie*-, but the meaning is rather generic and not clearly linked to the processing of cereals.

No further comparanda are at hand. While it seems tempting to compare Gk. πτύον, Att. πτέον n. ‘winnowing-shovel, fan’, as if from *(t)p(e)uH-*o*-, the initial cluster is not a regular development of PIE *p.

*rug^**h**^**-i-** (*rughi̯o- ‘Roggen’ [44:874]; *rughis [18:8, 432, 490]; *rughis / *rughyos [19:110–1]; *rughis ~ *rughyo- ‘rye’ [135:164]): Lith. *rugiaĩ*, Latv. *rudzi* m.pl. ‘rye’, OPru. EV *rugis* ‘Rocke’ < PB *rugi-; ORu. *rъžь*, Cz. *rež*, SCr. *rȃž* f. ‘rye’ < PSl. *rьžь; Proto-Permic *ruʒ́äg ≪ PIr. *ruǰa/ika-(?); ON *rugr* m. ‘rye’, OE *ryge* m. ‘rye’ < PGm. *rugi-; OFri. *rogga*, OS *roggo*, OHG *rocko* m. ‘rye’ < *ruggan-; MW *ryc*, W *rhyg* m. ‘rye’ <? PC *rukī/i̯o-

The Germanic and Balto-Slavic languages secure a North European formation *rug^*h*^-*i*- with the meaning ‘rye’. The British word may derive from divergent *rikī/i̯o- or *rukī/i̯o-, if not borrowed from Old English [19:110; 188:517]. The Thracian or Macedonian crop name βρίζα known from Galen and continued in the modern dialects in the sense ‘rye’ [9:128–9; 79:II, 265] is more probably connected with a different *Wanderwort* represented by Gk. ὄρυζα, Psht. *wríže*, Skt. *vrīhí*- ‘rice’ [104:283].

Importantly, forms resembling the European word appear in the Permic languages, cf. Ko. *ruʒ́e̮g*, Udm. *ʒ́eg*, dial. *ʒ́iźek*, which appear to continue Proto-Permic *ruʒ́äg. In view of the suffix *-*äg*, these have been adduced to substantiate a Proto-Iranian form *ruǰaka- or *ruǰika- [189], cf. Ko. *ide̮g* ‘angel’ ~ Oss. *(i)dawæg*, Ko. *ńebe̮g* ‘book’ ~ MP *nibēg*. No such form is attested anywhere in Iranian, however. Shu. (Bajui) *rōɣ̌ʒ* and Rosh. *růz* ‘ear (of grain)’ [20:222; 66; 190:876–7], adduced to substantiate PIIr. *ruȷ̌ika-, rather continue *rārza- [104:283; 145:67]. In view of the late adoption of rye in the Permic-speaking area, it has alternatively been suggested that the word was borrowed from an early Slavic dialect [191:3–4]. Given the presence of other Pre-Proto-Slavic loans in Ossetic [143], it is perhaps more plausible that the Slavic word was adopted by Iron Age steppe Iranian and from there permeated into Permic. Without a certain Iranian continuant, the word at any rate receives a (North) European distribution. In view of this areal range, it may be a late (dialectal) lexical innovation or—if the irregular Celtic form is to be relied on—a non-Indo-European *Wanderwort* [17:595].

The wild progenitors of rye spread from the Near East during the Neolithic, possibly as a weed infesting other grains. The transition of rye from a weed to a cultivated cereal is thought to have occurred in the Carpathian region from the second millennium BCE [192].

†*su(e)h_**2**_**-ro/eh**_**2**_**-** (*swaH_*2*_-*raH* [19:79–81]): Oss. I *xor* ‘grain, barley’, D *xwar* ‘grain, millet’; Lith. *sóros* f.pl. ‘millet’, Latv. *sûra*, obs. *sāre* ‘proso millet’

Witczak reconstructs (the equivalent of) *sueh_*2*_-*reh*_*2*_- ~ *suh_*2*_-*reh*_*2*_- for Baltic, but we are most probably dealing with a *Wanderwort* [47:29] also continued in Mordvin *suro* ~ *sura* ‘millet’ and perhaps Komi *ze̮r* ‘oats’ and Udmurt *ze̮r* ‘bromegrass’. The Ossetic word is rather derived from the Iranian root *hvar-, cf. YAv. *xvar*- ‘consume, eat’ < PIIr. *suar-, which is not consistent with a laryngeal.

?*tḱop-ero- (*k̂^*(*^*ó*^*)*^*pr̥* ‘oats’ [19:58; 193]):? Hitt. *kappar*- ‘greens(?)’ <? PAn. *ḱop-r-;? Jassic *zabar* ‘*avena*’ <? PIIr. *ć(š)ā̆para-; ON *hafri*, OS *haƀoro*, OHG *habaro*, *haparo* m. ‘oats’ < PGm. *hab(e/a)ran-

A protoform *ḱop-*ro*- meaning ‘oats’ has been reconstructed on the basis of Hittite, Germanic, Balto-Slavic and Iranian evidence [20:222; 104:282–3; 193; 194:133–43]. The comparison is invalidated by several issues. First of all, the meaning of Hitt. *kappar*- is unclear, which means that the proposed connection to PGm. *hab(a)ran- and Jassic *zabar*, both meaning ‘oats’, cannot be substantiated. Second, the Jassic form is only attested in a single word list and conspicuously close to Hung. *zab* ‘oats’, itself a borrowing from Sl. *zobъ [194:159]. It therefore is an exceptionally small basis for postulating a Proto-Indo-Iranian protoform *ćapara-, and a hypothetical Ossetic *sævær [88:III, 306]. If Yazg. *šebar* ‘Alpine sedge (*Carex nivalis*)’ is related, we must instead reconstruct PIIr. *ćiā̆parā̆- [195:301], or perhaps rather *ćšāpara- in view of Khot. *ṣavara*- ‘a green plant’, whose *ṣ* cannot reflect PIIr. *ć [104:283]. Neither of these reconstructions is compatible with the Hittite form. What is left is the (remote) possibility of connecting the Germanic and Iranian words, through a protoform *tḱop-*ero*-. If the Asian East Iranian comparanda are accepted, however, they would at the same time demonstrate that the meaning ‘(domesticated) oats’ is not necessarily old.

†*tḱor-iano- (*k̂þoryanos [19:99–100]): Arm. *cᶜorean* ‘wheat’; OIr. *tuirenn* f. ‘wheat’

A form *k̂þoryanos has been proposed by Witczak [19:99–100]. However, the productive Armenian suffix *-ean* cannot be equated with the Irish suffix *-enn* < PC *-*innā* ~ *-*indā* [123:199].

### 3.3. Exclusively European terms

?*g^**w**^**(e)u-s-o-** (*gēu-, *gəu*-, *gū*- [44:393–8]**)**: Lat. *būris*, *būra* f. ‘plow pole’ ≪? Sab. < PIt. *g^**w**^**euso-**; Gk. γύης m. ‘curved piece of wood in a plow’ < PGk. *guhā-

Lat. *būris* and *būra* are possible loans from Sabellic forms continuing PIt. *g^*w*^*euso-* [180:491–2; 196:321]. Assuming that Gk. γύης is a substantivization of the zero-grade root *g^*w*^*u*-, Latin and Greek would attest to two different ablaut grades of an original neuter *s*-stem *g^*w*^*éu*-*os*, gen. *gu-*s*-*és* ‘curve’ to the root *g^*(w)*^*eu*- ‘curve’. To further substantiate the Indo-European age of the *s*-stem, MP *gōšag* ‘corner, angle; detail’ [197:186] has been adduced, as if from a derived formation *g^*w*^*ou*-*s*-*o*- [180:321]. However, the better attested NP *gōšā* ‘angle, corner; handle of a vessel; loop, noose’ rather suggests an inner-Persian derivation from *gōš* ‘ear’ (cf. Sw. *öra* ‘ear; handle of a vessel’). In conclusion, even if the *s*-stem existed in Proto-Indo-European, its agricultural meaning may have arisen late in the Mediterranean branches.

*h_**2**_**eḱ-on-eh**_**2**_**-** (*ak̂en- [44:18–22]): Go. *ahana*, ON *ǫgn* f. ‘chaff’, OE *ægnan* f.pl. ‘awns, chaff, refuse’, OHG *agana* f. ‘chaff, awn, straw’ < PGm. *aganō-; Lat. *agna* f. ‘ear of grain’ < PIt. *akanā-

This is an Germano-Italic agricultural term. The formation resembles a neuter collective noun created to an *n*-stem *h_*2*_*eḱ*-*on*-, cf. the formally close Gk. ἀκόνη f. ‘whetstone’ as well as the potentially derived ἄκαινα f. ‘spike, prick, goad’ < *h_*2*_*eḱ*-*n*-*ih*_*2*_-. It appears that this formation went through a semantic shift from ‘sharp object’ to ‘awn’ in Germanic and Italic. The term additionally has a probable continuant in OPru. EV *ackons* ‘awn’, but the lack of palatalization of the velar is unexpected: if not due to depalatalization before *n* in one of the oblique cases [47:283], it could point to borrowing from Germanic, more specifically from a form similar to Go. *ahana*.

*h_**2**_**loh**_**1**_**-uo/eh**_**2**_**-** [79:8]: Gk. ἅλως, ἀλωή f. ‘threshing floor’ < PGk. *alōu̯(-ā)-; OSw. *lō* m. ‘threshing floor’ < PGm. *lō(w)a-

Two similar formations meaning ‘threshing floor’ are found in Germanic and Greek [168:78]. It is formally possible to connect the root *h_*2*_*elh*_*1*_- or *h_*2*_*leh*_*1*_- as found in Gk. ἀλέω ‘grind, bruise, mill’, Arm. *ałam* ‘grind’ and Av. *aša*- ptc. ‘ground’ [82:277], which also gave rise to the heteroclitic *h_*2*_*l(e)h*_*1*_-*ur/n*- continued by Gk. ἀλέατα ‘wheat-groats’, thematicized ἄλευρον, and possibly Arm. *alewr*, *aliwr* ‘flour’, if this is not a Greek loan [198:90–5].

An additional (or alternative?) cognate may be found in Hitt. *ḫall*-*anna*-^*i*^ ‘trample down, flatten (fields and plants)’. This verb apparently constitutes an imperfective formation in -*anna/i*- to the etymologically obscure root *ḫall- < *h_*2*_*(e)lH*- or *h_*3*_*(e)lH*- [75:271]. If related, an Indo-Anatolian root *h_*2*_*elh*_*1*_- or *h_*2*_*leh*_*1*_- ‘flatten’ can be postulated, which in core Indo-European became associated with an activity related to the processing of cereals, possibly the technique of threshing cereals by having animals tramp them. It is more difficult to explain the emergence of the meaning ‘grind’ from the same semantic specialization, however.

*(H)lois-eh_**2**_**-** (*loisā ‘Furche’ [44:671]): OCS *lěxa* f. ‘row’, Ru. dial. *lexá*, *léxa* f. ‘furrow, bed’, SCr. *lĳèha* f. ‘plot, ridge, flower bed’ < PSl. *lěxa; OHG -*leisa* f. ‘track’ < PGm. *laisō-; Lat. *līra* f. ‘furrow’ < PIt. *loisā-

This is an *eh*_*2*_-stem derived from an obscure root *(H)leis- [82:209], probably with an original meaning ‘track, trace’, but with agricultural associations in at least Italic and Slavic. The appurtenance of Lith. *lýsė* f. ‘bed (garden)’ (and by extension OPru. EV *lyso* ‘bed (field)’) seems likely, but the acute intonation is problematic in that it at face value points to *(H)liH-*s*-.

?*k^**w**^**eh**_**2**_**t-i-** (?*k^*w*^*et*- [18:104]):? Gk. Hes. πήτεα ‘bran’; OIr. *cáith* f. ‘chaff, husks’ < PC *k^**(w)**^**āti-**

The Celtic and Greek forms are suggestive of an *i*-stem *k^*w*^*eh*_*2*_*t*-*i*-. This *i*-stem may be derived from a verbal root *k^*w*^*eh*_*2*_*t*-, as found in Gk. πάσσω ‘strew, sprinkle’ and Lat. *quatiō* ‘shake’ < *k^*w*^*h*_*2*_*t*-*ie*- [168:1155], and potentially Lat. *quālus* m. ‘wicker (*winnowing?) basket’, if from PIt. *k^*w*^*h*_*2*_*t*-*slo*- [48:504]. However, the marginal attestation of Gk. πήτεα as a Hesychian gloss detracts from the feasibility of the comparison, particularly given the attribution of the derived formation πητ[ε]ῖται ‘bread made with bran’ to Laconian, where PGk. *ā ought to have been preserved. The reconstruction *k^*w*^*eh*_*2*_*t*-*i*- is thus rendered uncertain [44:632]. The alternative reconstruction of the root as a root *(s)ku̯eh_*1*_*t*- [82:563] can only be reconciled with OIr. *cáith* by starting from an isolated *o*-grade form *ku̯oh_*1*_*t*-*i*-.

†*mel(H)-i- (*mél-*i*-, -*n*-*és* [44:718];? *melh_*2*_- [18:383]; *melH-*i* [19:77]): Lat. *milium* n. ‘millet’ < PIt. *melio-; Gk. μελίνη f. ‘millet, esp. foxtail’ < PGk. *melinā-;? Lith. *málnos* f.pl. ‘sweetgrass’ < PB? *malʔna-

It is unclear if all the forms belong together due to the difference in vocalism and suffixation. They have been explained as a heteroclitic *i*/*n* stem [44:718; 48:379], which would make them highly archaic. However, the appurtenance of Lith. *málnos* f.pl. ‘floating sweetgrass (*Glyceria fluitans*)’ is doubtful: Lith. *málna* must rather be a loan from Polish *manna* ‘floating sweetgrass’ (cf. German *Mannagras*, *Mannaschwaden*) [199:167–8], with dissimilation of the geminate /nn/ to /ln/. Lat. *milium* ‘millet’ is often derived from PIt. *meli-, with quasi-regular raising of *e* to *i* before *i in the following syllable [200:81]. The same *i*-stem has been argued to be behind Gk. μελίνη. The reconstruction of a shared *i*-stem is uncertain, however, since Lat. *milium* is synchronically a *io*-stem (cf. Skt. *sasyá*-, PGm. *hersja-, etc.) and Gk. μελίνη a substantivization of an adjective in -*ino*-.

As to a root etymology for the Latin and Greek forms, the most popular suggestion is a connection with *melh_*2*_- ‘grind’ [44:718; 79:374; 83:I, 88]. An alternative is a connection with Gk. μέλας ‘black’ paralleled by e.g. Skt. *śyāmā́ka*- ‘a type of millet’ and Fr. *millet*/*blé noir* ‘buckwheat’ [19:77; 201:113]. Regardless of the identity of the root, Lat. *milium* and Gk. μελίνη look like independent formations, meaning that no shared Indo-European protoform can be given.

Other comparanda, such as Khow. *blan* ‘species of barley’ < PIIr. *mlāna- and ON *melr* m. ‘sand ryegrass (*Elymus arenarius*)’ [19:77], are even more doubtful. ON *melr* is short for the compound continued as Icel. *mel*-*gresi*, whose first element can be identified as ON *melr* m. ‘sandbank’ < PGm. *melha- [202:615]. Khow. *blan* has alternatively been derived from an isolated Indo-Iranian root, cf. Skt. *mlā*- ‘to be limp, wither’ [133:599], possibly a variant of *marH- ‘to crush’ [77:II, 388].

?*ne(h_**1**_**)i-uo-** (*nei-*u̯o*- [44:311–4; 79:8]): Gk. νειός f. ‘fallow land’; OCS *n’iva*, Ru. *níva*, SCr. *njȉva* f. ‘(arable) field’ < *njìva

This old comparison involves several phonological problems. The Greek word may be derived from PIE *ni ‘low, below’, through an adjective *nei-*uo*- ‘low-lying’, or from *neu-*io*- ‘new ground’. The Slavic form *njiva can regularly continue *njūva, which has been considered a contamination of the PSl. outcomes of *neu-*h*_*2*_ and a zero-grade form *nu-*h*_*2*_ [47:303–4]. While it is possible to explain both formations as continuations of a neuter *u*-stem *né(h_*1*_*)i*-*u*, gen. *n(h_*1*_*)i*-*éu*-*s*, pl. *né(h_*1*_*)i*-*u*-*h*_*2*_, the many formal ambiguities cast doubt on the validity of the comparison.

*neik- (*neik- ‘Getreide schwingen’ [44:761]): Lith. *niekóti*, Latv. *niẽkât* ‘winnow’ < PSl. *ne/aik-; Gk. λίκνον, νίκλον n. ‘winnowing fan’ < PGk. *niklo- or *nikno-; OSw. *nēk* f. ‘sheaf’ < PGm. *naikō-; MIr. *cruth*-*necht* f. ‘wheat’, MW *gwenith* ‘id.’ < PC *-nixto-

A root *neik- is reconstructed on the basis of Celtic, Greek and Baltic. Arm. *nk‘oyr* ‘sieve’ is also sometimes compared [128:III,477], but -*oyr* as a nominal suffix is hard to explain. It does seem possible to adduce PGm. *naikō-, which through Pre-Proto-Germanic *naikkā- can regularly continue *noik-*néh*_*2*_- (with Kluge’s law). Together, these formations secure a shared agricultural meaning ‘winnow’ for most of the European branches. Given the regular sound correspondences across the branches, there is no reason to doubt the inherited character of the word [*pace* 47:303–4].

On a deeper level, it seems likely that the root in question originated as a (core Indo-European?) semantic specialization of an Indo-Anatolian root *neik- ‘raise, stir up’, cf. Hitt. *nini(n)k*-^*zi*^ ‘set in motion, raise, stir up’, Gk. νεῖκος ‘quarrel, strife’, RuCS *niknuti*, SCr. *nȉknuti* ‘appear, arise’. Suitable semantic parallels are at hand, cf. Skt. *úd*-*bharati* ʻraiseʼ vs. Marathi *ubharṇẽ* ʻwinnow’ [133:75] and Skt. *ut*-*phalati* ʻspring open, jump outʼ vs. Marathi *uphāḷṇẽ* ʻwinnowʼ < *ut-*phālayati* [133:84].

*polḱ-eh_**2**_**-** (*polk̂ā ‘Gewendetes’ [44:807, 850]; *polk̂éh_*a*_ ‘± fallow land’ [18:200]):? ORu. *polosa* f. ‘strip of land’, Cr. dial. *plȁsa* f. ‘treeless land’ < PSl. *polsa; OE *fealg*, MDu. *valghe*, G Bav. *Falg* f. ‘fallow land’ < PGm. *falgō-; LLat. *olca* ‘fertile field’, Fr. *ouche* f. ‘plantation; arable field’ ≪ Gaul. *olca < PC *ϕolkā

A marginally attested formation found in at least Germanic and Celtic, although the latter is only indirectly recorded through Romance. The Slavic form has alternatively been derived from *polH-*o*- ‘field’ [47:288].

*prḱ(-eh_**2**_**)-** (*pr̥k̂ā ‘Furche’ [44:821]; *pŕ̥k̂eh_*a*_ [18:215]): ON *for* f. ‘rivulet; mud’, OE *furh*, *fyrh* f. ‘furrow’, OHG *furh*, *furuh* f. ‘furrow’ < PGm. *furh-; Lat. *porca* f. ‘furrow’ < PIt. *porkā; Gaul. *rica*, W *rhych* f. ‘furrow’ < PC *ϕrikā

A West European term for ‘furrow’. Within Germanic, it is in ablaut relation with Nw. dial. *fere* m. ‘strip or plot of land; ridge between furrows’ < *ferhan- [203:244]. Further possible cognates, but without agricultural semantics, are Lith. *pró*-*perša* ‘thawed patch in the ice, gap (in the clouds)’, Skt. *párśāna*- m. ‘chasm(?), valley(?)’, which may ultimately derive from a verbal base ‘dig, tear’ [82:475]. This allows us to assume the word originally had a sense ‘rift, gap’, which apparently acquired an agricultural use in the European centum languages. Formally, the Germanic root noun can be separated from the *eh*_*2*_-stem found in Italic and Celtic.

*seǵ^**h**^**-e-tleh**_**2**_**-** (*seĝhedhlā [44:888–9]): Gk. ἐχέτλη f. ‘plow handle’ < PGk. *sek^**h**^**etlā**; OW *edil* gl. *stipa*, W *haeddel* f., MBret. *haezl*, MoBret. *hael* ‘plow handle’ < PBr. *sagetlā <? PC *segetlā

Both the Greek and Brittonic words have been derived from (a thematic stem of) the root *seǵ^*h*^- ‘hold firmly’ (cf. Gk. ἔχω ‘have, hold’) [180:495]. The Brittonic material appears to require an onset *saǵ^*h*^- while the Greek requires *seǵ^*h*^-. Pokorny offers a now-obsolete PIE reconstruction whereby a reduced vowel *s_*e*_*ǵ*^*h*^- was vocalized into *a in Celtic [44:889]. Hamp departs from a *sǵ^*h*^-*e*- that received a ‘prop vowel’ and independent vocalization with *e in Greek [204–206]. Schrijver more convincingly takes *seǵ^*h*^- to be original and proposes a Brittonic sound law PC *e > (*æ >) PBr. *a before *ge, *gi [51:134–41].

The W ending -*ddel* for PIE *-tleh_*2*_ is unexpected: the regular outcome of *-tleh_*2*_ is W *-dl*, cf. *anadl* ‘breath’ < *h_*2*_*enh*_*1*_-*tleh*_*2*_. This leads Pokorny and Hamp to reconstruct its allomorph *-*d*^*h*^*leh*_*2*_ > *-θλη that was dissimilated to -τλη following a voiced aspirate in the stem. However, it is likely that both *-*d*^*h*^*lo/eh*_*2*_ and *-*tlo/eh*_*2*_ merged into *-*ðl* in a Common Brittonic stage [51:363]. Thus the most parsimonious reconstruction is *-*tleh*_*2*_.

As a result, an exact Greco-Celtic isogloss for ‘plow handle’ may tentatively be reconstructed.

*seh_**1**_**-men-** (*sē-*men*- ‘Samen’ [44:889–91]; *seh_*1*_*mn̥* [19:118]): Lith. *sėmenys* pl. ‘linseed’, OPru. *semen* ‘seed’ < PB *seʔ-men-; OCS *sěmę* n. ‘seed’ < PSl. *sěmę; OS, OHG *sāmo* m. ‘seed’ < PGm. *sēman-; Lat. *sēmen* n. ‘seed’ < PIt. *sēmen-

This is an *mn*-stem derived from the root *seh_*1*_- ‘sow’. It has been argued that the meaning ‘sow’ developed from ‘put in (the ground)’, cf. Hitt. *šai*-^*i*^ ‘impress, prick’ < *sh_*1*_-*oi*- [125:504], in core Indo-European. This would be a clear *terminus post quem* for the creation of this *mn*-stem. However, if Hitt *šēli*- ‘granary’ is to be compared to OIr. *síl* ‘seed’ through a protoform *seh_*1*_-*li*- [75:743–4; 207:541], the meaning ‘sow’ must already have been present in Indo-Anatolian [208:167], even if it was part of a wider semantic range.

*solk-o- (*solko-*s* ‘Zug’ [44:901]): Gk. ὁλκός m. ‘hauling-engine; furrow, track; ditch, channel’ < *holko-; Lat. *sulcus* m. ‘furrow’ < PIt. *solko-

Two formations in the European centum languages derived from the root *selk- ‘draw’, cf. ToB *sälk*- ‘pull, draw’, Gk. ἕλκω ‘draw, drag’ and probably also Arm. *hełg* ‘lazy, slow’, exhibit a semantic shift to ‘draw furrows, plow’: 1) an *o*-stem shared between Latin and Greek, and 2) an isolated root noun in Germanic, viz. OE *sulh* f. ‘furrow; plow’ < PGm. *sulh-. The semantic shift appears complete in Germanic and Italic, whereas a more original range of meanings remains in Greek.

*spor-eh_**2**_**-** (*sporáH_*2*_ [19:119]): Gk. σπορά f. ‘seed’; Alb. *farë* f. ‘seed, sperm’ < PAlb. *farā

This formation, clearly derived from the PIE root *sper-, appears shared between Greek and Albanian [63:56]. The original meaning of the PIE root *sper- was ‘scatter’, cf. Hitt. *išpār*-^*i*^ ‘spread (out), strew’, which developed into ‘sow’ in some of the European branches, cf. Gk. σπείρω ‘scatter, spread; sow’, whence also Gk. σπέρμα ‘seed, sowing’ and σπόρος ‘seed’. In Celtic, the isolated Bret. (Pelletier) *fer* ‘lentils’ could theoretically continue another formation derived from the root *sper-, but because of the absence of cognates in British or Goidelic, this cannot be verified.

*uog^**wh**^**-(m)nis-** (*u̯og^*w*^*hni*-*s*, *u̯og^*w*^*hnes*- ‘Pflugschar’ [44:1179–80]): Gk. Hes. ὀφνίς ‘plowshare; plow’ < PGk. *u̯ok^**wh**^**(s)ni(s)-**; OPru. *wagnis* or *wagins* ‘coulter’ < PB *wagnV-; ON *vangsni*, OHG *waganso*, *wagi(n)so* m. ‘plowshare’ < PGm. *wagnisan-; Lat. *vōmer* m. ‘coulter, plowshare’ < PIt. *woχ^**w**^**-(s)mis-(?)**

A well-known European word, possibly derived from a root *ueg^*wh*^- [44:1179–80] as apparently found in Lith. *vagà* f. ‘groove, furrow; patch of arable land’ < *uog^*wh*^-*eh*_*2*_- and ON *veggr*, OE *wecg*, OHG *wecki* ‘wedge’, Lith. *vãgis*, Latv. *vadzis* ‘peg; wedge’ < *uog^*wh*^-*io*- [44:1179–80; 47:297; 55:1581–3].

The original suffixation appears to have been *-*nis*-, as this might be the common denominator for at least Greek and Germanic. In latter branch, at least ON *vangsni* looks like it could continue *wagnisan-. OHG *waganso*, on the other hand, was probably influenced by *alansa* f. ‘awl’ and *segansa* f. ‘scythe’, whose suffixes appear to have metathesized from *-*es*-*neh*_*2*_- [209:29].

The interpretation of the Italic and Baltic forms is more difficult. In Italic, the *m* of the suffix is unexpected and requires a phonetically conceivable but *ad hoc* assumption that it was rounded by the preceding labiovelar. Alternatively, it is possible to assume that all forms originally contained *-*mn*- and were reduced differently [180:491]. Concerning the Baltic evidence, Smoczyński reads the Prussian word as <wagins> and analyzes it as a Germanic loan (MHG *wagense*) [210:132–3]. While this cannot be ruled out, Fi. *vannas* and Est. dial. *vadnas* ‘plowshare’ (< *vatnas) would be most easily explained as a loan from Baltic [211].

An early plowshare made of deer-antler has been found in phase B of the Gumelniţa culture [212; 213] dated to the mid-4th millennium BCE. In Yamnaya contexts, a triangular sandstone from the Mikhailovka culture is interpreted as having been used as one [214:161]. Indo-European speakers may have become acquainted with this tool in these particular areas.

## 4. Results

### 4.1. Evaluation of the data

From the evaluation of the data presented here, which consists of cereal (cultivation and processing) terms with cognates in at least two independent Indo-European branches, several conclusions can be drawn.

First of all, strict application of the known sound laws has revealed that many of the previously proposed comparisons, including some listed by Mallory [10], are formally problematic. The formal problems are of a diverse nature. In many cases, reconstructions were in need of revision. We have, for instance, modified *ǵ^*h*^*ersd*^*h*^- to *ǵ^*h*^*ersd*-. This is the least problematic category, however, as minor formal corrections are typically inconsequential to whether a term was inherited or not in the branches in which it occurs. In other cases, cognates had to be removed from the cognate set, leading to a more limited distribution in the Indo-European language family and potentially a more shallow time depth. Here we may mention removed cognates such as Hitt. *šēša*-, which cannot regularly be derived from *se-*sh*_*1*_-*o*-. It is particularly striking that in many cases, material from the Iranian languages has been liable to misinterpretation, probably due to their relatively late attestation and opaque evolution. Notable here is NP *zurd* ‘millet’ as a false continuant of *ǵ^*h*^*ersd*-. Where formal problems were insurmountable, comparisons had to be given up entirely, leading to a more radical reduction of the corpus of potentially inherited lexical items. Examples of such rejected comparisons are *keres- and *pano-, both assumed to have referred to millet. Strikingly, not a single word for millet can be reconstructed for Proto-Indo-European.

Special attention is required for terms showing resemblances that appear undeniable, but nevertheless exhibit irregular sound correspondences, and in addition have a localized or areal distribution, e.g. limited to (parts of) Europe. When the protoforms of the branches involved cannot be unified into a single reconstruction, the comparanda may indicative of prehistoric borrowing processes, i.e. reflect different manifestations either of an old *Wanderwort* or of a term borrowed from a lost, non-Indo-European language (group). Accordingly, at least two terms have been reclassified from the inherited, potentially Indo-European category into a category of prehistoric loans from one or more unknown sources: *b^*h*^*ars*- ‘a cereal’ and *au̯iĝ- ‘oats’. Neither of these traditional reconstructions can be maintained for any level within the Indo-European pedigree.

Beside the many formal problems, the reconstruction of the meanings often appears problematic. For a start, many of the proposed etymologies have been overinterpreted semantically, i.e. they have been assigned an agricultural meaning while in fact no such meaning is evident for the Proto-Indo-European level. In many cases, an agricultural meaning is present in some of the cognates, but not all of them. The formation *d(e)rH-*ueh*_*2*_-, for instance, refers to a kind of grass in Indic and Celtic, and to wheat only in Middle Dutch. As a limited distribution of an agricultural meaning is most easily understood as resulting from an equally limited, post-Indo-European innovation, those meanings should not uncritically be projected back into the protolanguage. In many cases, it can be demonstrated that a meaning associated with the cultivation and processing of cereals does not date back to the oldest strata of the family, but developed at more shallow stages in a subset of the Indo-European branches. For instance, counter to previous views (see Table 1), the Proto-Indo-European meaning of *ǵrH-*no*- was not ‘cereal’, but rather ‘granule’, a meaning still extant in Germanic and Italic. Likewise, the Proto-Indo-European meaning of *pelH-*u*- cannot have been ‘chaff’; this meaning is dominant only in Balto-Slavic and Indo-Iranian, but the other branches in which the word occurs rather have ‘dust’, ‘powder’ or even ‘snow’. By contrast, the term *puH-*ro*- does refer to a cereal in all the branches in which it occurs. However, this formation was probably derived from the root *peuH- ‘clean, purify’, which could not have happened before this root acquired the secondary meaning ‘winnow’. And while the semantic shift from ‘clean, purify’ to ‘winnow’ is indeed visible in Indo-Iranian, it does not seem to have spread to the West European centum languages. Apparently, this shift, too, was of post-Proto-Indo-European date.

Intriguingly, it is evident that many agricultural meanings that have habitually been reconstructed for Proto-Indo-European are effectively post-Anatolian. This has previously been demonstrated for the root *h_*2*_*erh*_*3*_-, meaning ‘crush, shatter’ in Anatolian, but ‘plow’ in core Indo-European, including Tocharian. The root *sper- means ‘scatter’ in Anatolian, but displays a semantic shift to ‘seed’ in Greek and Albanian. The core Indo-European root *h_*2*_*leh*_*1*_- ‘grind; thresh’ could be the continuation of what in Hittite appears as *ḫall*- ‘tramp(le), flatten’. It is further attractive to assume that the root *neik-, meaning ‘winnow’ in a large subset of the European branches, is etymologically identical to the root *neik- ‘raise, stir’, already found in Hittite. Even younger are the meanings that are of post-Tocharian date. Here we can mention the well-known example of *g^*w*^*r(e)h*_*2*_-*uon*-, meaning ‘stone’ in Tocharian, but ‘grindstone’ in the other branches in which it is attested. In addition, there is the *s*-stem *h_*2*_*eḱ*-*os*- meaning ‘tip (of grass)’ in Tocharian, and ‘ear (of grain)’ in Germanic and Italic only.

It is, moreover, especially striking that several instances of semantic specialization are found exclusively in the European centum languages. The root *selk- ‘draw’, as continued by ToB *sälk*- and Gk. ἕλκω, served as the basis for a root noun *slk- ‘furrow; plow’ in Germanic and an *o*-stem *solk-*o*- ‘furrow’ in Italic and Greek. The *s*-stem *h_*2*_*eḱ*-*os*- meaning ‘tip (of grass)’ in Tocharian, acquired the meaning ‘ear (of grain)’ in Germanic and Italic only. The related collective formation *h_*2*_*eḱ*-*on*-*eh*_*2*_- similarly acquired an agricultural meaning in the same branches. These semantic shifts, often absent or marginal in Greek, appear to cluster in the West European centum languages, and—if not independent—must have appeared late, in an already fragmenting, core Indo-European dialect continuum. A complete overview of the semantic intricacies of the various terms is given in Table 2.

**Table 2:** Overview of cereal cultivation and processing terms that conform to the known sound laws and have cognates in at least two Indo-European branches.

formal reconstruction	(original) generic meaning	Anatolian	Tocharian	Indic	Iranian	Armenian	Greek	Albanian	Baltic	Slavic	Germanic	Italic	Celtic	(derived) agricultural meaning
*d(e)rH-ueh_2_-	wild grass	-	-	+	-	-	-	-	†	-	+	-	+	wheat
*d^h^oH-neh_2_-	seeds	-	?	+	+	-	-	-	+	-	-	-	-	cereal
*ǵ^h^elH-	cut	-	-	-	-	?	-	-	-	-	+	-	+	plow
└›?*ǵ^h^olH-o-	stick	-	-	?	-	+	-	-	-	-	-	-	-	ard?
*ǵ^h^ersd-		?	-	-	†	-	-	?	-	-	+	+	-	cereal
*ǵ^h^reud-	crush	-	-	-	-	-	-	-	+	-	+	-	-	crush grains
└›*ǵ^h^rud-o(n)-		-	?	-	-	-	-	†	+	-	+	-	-	cereal
*ǵrH-no-	granule	-	-	-	?	-	-	-	+	+	+	+	?	cereal
?*g^w^eu-os-	curve	-	-	-	?	-	+	-	-	-	-	?	-	plow pole
*g^w^r(e)h_2_-uon-	stone	-	+	+	?	+	-	-	+	+	+	-	+	pestle, quern
*h_2_ed-	dry, parch	+	-	-	?	-	+	-	-	-	-	-	-	roast grains?
└›*h_2_ed-o(s)-		-	-	-	-	+	-	-	-	-	+	+	?	cereal
*h_2_eǵ-ro-	plain	-	-	+	+	-	+	-	-	-	+	+	-	field
*h_2_e(h_2_)i-r-ieh_2_-	wild grass	-	-	?	-	-	+	-	+	-	-	-	-	darnel
?*h_2_eḱ-ti-	tip	-	?	-	-	-	-	-	+	+	-	-	†	ear
*h_2_eḱ-on-eh_2_-	spike	-	-	-	-	-	+	-	?	-	+	+	-	awn
*h_2_eḱ-os-	tip (of grass)	-	+	-	-	-	-	-	-	-	+	+	-	ear
*h_2_(e)lb^h^-it-		-	-	-	†	-	+	+	-	-	-	-	-	barley
*h_2_erh_3_-	crush	+	+	-	-	-	+	-	+	+	+	+	+	plow
└›*h_2_erh_3_-tro-		-	+	-	-	+	+	-	+	+	+	+	+	plow
└›*h_2_erh_3_-ur/n-		-	-	+	+	+	+	-	-	-	-	-	+	field
*h_2_leh_1_-	flatten?	?	-	+	+	+	+	-	-	-	-	-	-	crush grains
└›*h_2_l(e)h_1_-ur/n-		-	-	-	-	+	+	-	-	-	-	-	-	flour
└›*h_2_loh_1_-uo/eh_2_-		-	-	-	-	-	+	-	-	-	+	-	-	threshing floor
*Hoket-(i)eh_2_-		-	-	-	?	-	-	-	+	-	+	+	+	harrow
*(H)ieu(H)-		+	-	+	+	?	+	-	+	+	-	-	-	cereal
*k^w^eh_2_t-	shake	-	-	-	-	-	+	-	-	-	-	?	-	sieve?
└›?*k^w^eh_2_t-i-		-	-	-	-	-	?	-	-	-	-	-	+	chaff
?*k^w^els-	drag	-	-	+	+	-	?	-	-	-	-	-	-	plow
*^(^ḱ^)^eh_2_p-o/eh_2_-		-	-	-	?	-	+	?	-	-	+	-	-	plot of land
*(H)lois-eh_2_-	track	-	-	-	-	-	-	-	-	+	+	+	-	furrow
*neik-	stir up	+	-	-	-	-	+	-	+	-	+	-	+	winnow
*peis-	rub	?	-	+	+	-	+	-	+	+	+	+	+	grind (grain)
*pelH-	sprinkle	-	-	-	-	-	+	+	-	-	+	+	-	sieve?
└›*pelH-ou-	dust	-	-	+	-	-	-	-	+	+	-	?	-	chaff
└›*p(o)lH-u-	dust	-	-	-	-	-	+	+	-	-	+	?	-	flour
?*perḱ-	dig, tear	-	-	+	-	-	-	-	+	-	-	-	-	plow?
└›*prḱ(-eh_2_)-		-	-	-	-	-	-	-	-	-	+	+	+	furrow, balk
*peuH-	clean	-	-	+	-	-	-	+	-	-	+	+	+	winnow
└›*puH-ro-		-	-	†	-	-	+	-	+	+	-	-	-	cereal
*polḱ-eh_2_-		-	-	-	-	-	-	-	-	+	+	-	+	arable land
*rug^h^-i-		-	-	-	?	-	-	-	+	+	+	-	?	rye
*seǵ^h^-e-tleh_2_-		-	-	-	-	-	+	-	-	-	-	-	+	plow handle
*seh_1_-	impress	+	-	-	-	-	-	-	+	+	+	+	+	sow
└›*seh_1_-men-		-	-	-	-	-	-	-	+	+	+	+	-	seed
└›*se-sh_1_-io-	seeds	†	-	+	+	-	-	-	-	-	-	-	+	cereal
*selk-	draw	-	+	-	-	+	+	-	-	-	+	-	-	plow
└›*solk-o-		-	-	-	-	-	+	-	-	-	-	+	-	furrow
*serp-	cut	-	-	-	-	-	-	-	-	-	+	+	-	prune
└›*serp-o/eh_2_-		-	-	†	-	-	+	-	+	+	-	-	?	sickle
*sper-	scatter	+	-	-	-	-	+	-	-	-	-	-	-	sow
└›*spor-eh_2_-		-	-	-	-	-	+	+	-	-	-	-	-	seed
*uers-	sweep	+	-	-	-	-	-	-	+	+	+	+	-	thresh
*uog^wh^-(m)nis-		-	-	-	-	-	+	-	+	-	+	+	-	plowshare

The reliability of the cognates in the branches is indicated with the symbols + (present),? (possibly present) and—(absent). Branches for which a cognate has been rejected receive a dagger. Coloration indicates whether the agricultural or non-agricultural semantics, as given to the left and right, are present in the involved branch: green: exclusively agricultural; yellow: both agricultural and non-agricultural; orange: exclusively non-agricultural.

Evidently, many of the formal and semantic issues tie back into the problem of the phylogeny of the Indo-European languages. In the starburst model, in which all core Indo-European branches are treated as equally distantly related, a term shared by as few as two branches must be admitted to the protolanguage, whereas a more structured model allows for more strata. Our findings underline that the latter is a priori more realistic than the starburst model. The creation of terms shared only by a limited subsection of Greek and Albanian, e.g. *h_*2*_*(e)lb*^*h*^-*it*- ‘barley’ and *spor-*eh*_*2*_- ‘seed’, may be as recent as the last common ancestor, and should not be projected back into Proto-Indo-European, let alone Proto-Indo-Anatolian, at least not without the strongest of reservations. Furthermore, the demonstrable presence in our findings of formal and semantic archaisms in Anatolian and to a lesser extent in Tocharian unquestionably supports the modern consensus that these branches diverged from the other, core Indo-European branches relatively early. It appears that the split between basal and core Indo-European is more fundamental than the split between the European and Asian branches, at least in this subsection of the lexicon.

In conclusion, while many cereal terms have been proposed in the literature, their number must be substantially reduced, especially for the most basal stage of Indo-European, Indo-Anatolian. The resulting picture is one that is far less problematic to the Steppe Hypothesis than has been previously suggested [10]. The overall scarcity of shared cereal (cultivation and processing) vocabulary at this stage strongly contradicts a deeply agricultural language community and thus disqualifies the Anatolia Hypothesis as it was initially formulated. The results in fact also contradict the revised form of the hypothesis, which entailed a scenario in which core Indo-European was introduced to the Pontic-Caspian steppe by an outmigration from an agrarian homeland in Anatolia. This scenario implies that Indo-Anatolian was originally rich in agricultural vocabulary, but that this part of the lexicon was largely lost in core Indo-European during an economic transformation from sedentary farmers to mobile pastoralists. The linguistic evidence is suggestive of the opposite scenario in which core Indo-European repurposed various originally non-agricultural Indo-Anatolian lexical roots to reference an increasingly agricultural economy.

Nevertheless, our results also raise questions for the Steppe Hypothesis. For the oldest stratum, Indo-Anatolian, the lexical evidence for cereal use is relatively modest, but not zero: we must at least admit the cereal term *(H)ieu(H)- and perhaps *ǵ^*h*^*(e)rsd*-. For the core Indo-European level, an even more extensive set of terms can be identified. In a model in which the split between the European and Asian branches is assumed to be primary, we must admit at least *h_*2*_*erh*_*3*_- ‘plow’, *h_*2*_*erh*_*3*_-*ur/n*- ‘(arable) field’, *peis- ‘grind (grain)’, *se-*sh*_*1*_-*io*- ‘a cereal’, *h_*2*_*ed*-*o(s)*- ‘a (parched?) cereal’, *d^*h*^*oH*-*neh*_*2*_- ‘(cereal) seed’ and *pelH-*u*- ‘chaff’. By applying the alternative, Indo-Slavic model, it is possible to relegate the latter two terms to the most recent subnode of the family, so as to deprive them of their core Indo-European status. However, even in this model, the remaining terms still stand. It is furthermore worth noting that at the second-most basal stage, prior to the Tocharian split, the root *h_*2*_*erh*_*3*_- had already undergone the semantic shift to ‘plow’, implying that this practice was known to the deepest layers of core Indo-European. In other words, unless cereal cultivation was a much more important aspect of the Yamnaya culture than recent archaeological interpretations suggest, this culture does not offer a perfect archaeolinguistic match for the original language community of the core Indo-European branches, including Tocharian. As a consequence, we may conclude that it is not possible to on the one hand support the Steppe Hypothesis (or the revised Anatolia Hypothesis for that matter) while at the same time assuming that steppe migrants had an exclusively pastoralist way of life, as has been proposed for the early Yamnaya culture [41; 42; 215:17].

### 4.2. The position of Indo-Iranian: Hirt vs Schrader

We shall now return to the age-old question of to what extent Indo-Iranian participated in the general shift of the core Indo-European subgroups from a largely pastoralist economy to a more agricultural way of life. The question revolves around the two rival hypotheses by Hirt on the one hand and Schrader on the other: did Indo-Iranian lose many of the agricultural terms present in the European branches or did the European branches rather acquire them after the Indo-Iranian split?

As described above, multiple semantic innovations can be observed in the European languages. Many of these innovations appear late and dialectally limited, i.e. post-Tocharian at the earliest and pan-European at best. They demonstrate how the European Indo-European dialects, in the period when they had started diverging from each other, were in the process of repurposing the vocabulary they had inherited from basal and core Indo-European to reference an increasingly agricultural way of life. However, Indo-Iranian typically does not participate or only marginally participates in the semantic shifts that characterize the European branches. This is evinced by a number of very subtle archaisms in this branch. An association of *ǵrH-*no*- ‘granule’, plausibly derived from a root *ǵerH- ‘scatter’, with domesticated plant seeds is visible in Germanic, Ital(o-Celt)ic and Balto-Slavic, but if Pashto *zə́ṇai* is to be relied on, (Indo-)Iranian may have preserved a more general meaning, i.e. a seed of any (domesticated or non-domesticated) plant. The root *peuH- retained its original meaning ‘purify’ in Germanic, Celtic and Italic. It might have developed into ‘winnow’ in Balto-Slavic, Greek and possibly Albanian, in view of the derivation *puH-*ro*- ‘a kind of cereal’, but Indo-Iranian takes up an intermediate position, in that it preserves the polysemy. Grinding is an activity that is not restricted to agricultural societies. Nevertheless, it is striking that the formation *g^*w*^*r(e)h*_*2*_-*uon*- has the generic meaning ‘stone’ in Tocharian, the more agricultural meaning ‘quern’ or ‘millstone’ in Germanic, Celtic, Armenian and Balto-Slavic, but the semantically intermediate ‘(pressing) stone’ in Sanskrit. A final showcase exemplifying the comparatively archaic semantics of Indo-Iranian is that of PIE *h_*2*_*eǵ*-*ro*-, whose original meaning ‘plain (for driving cattle?)’ was preserved in Indo-Iranian, while the European branches Germanic, Italic and Greek share a (partial) semantic shift to ‘cultivated field’ [79:9]. Although often subtle, at least some of these differences in meaning attest to unidirectional semantic shifts in the European branches towards a more agricultural way of life to the exclusion of the Indo-Iranian branch.

**Fig 2:**
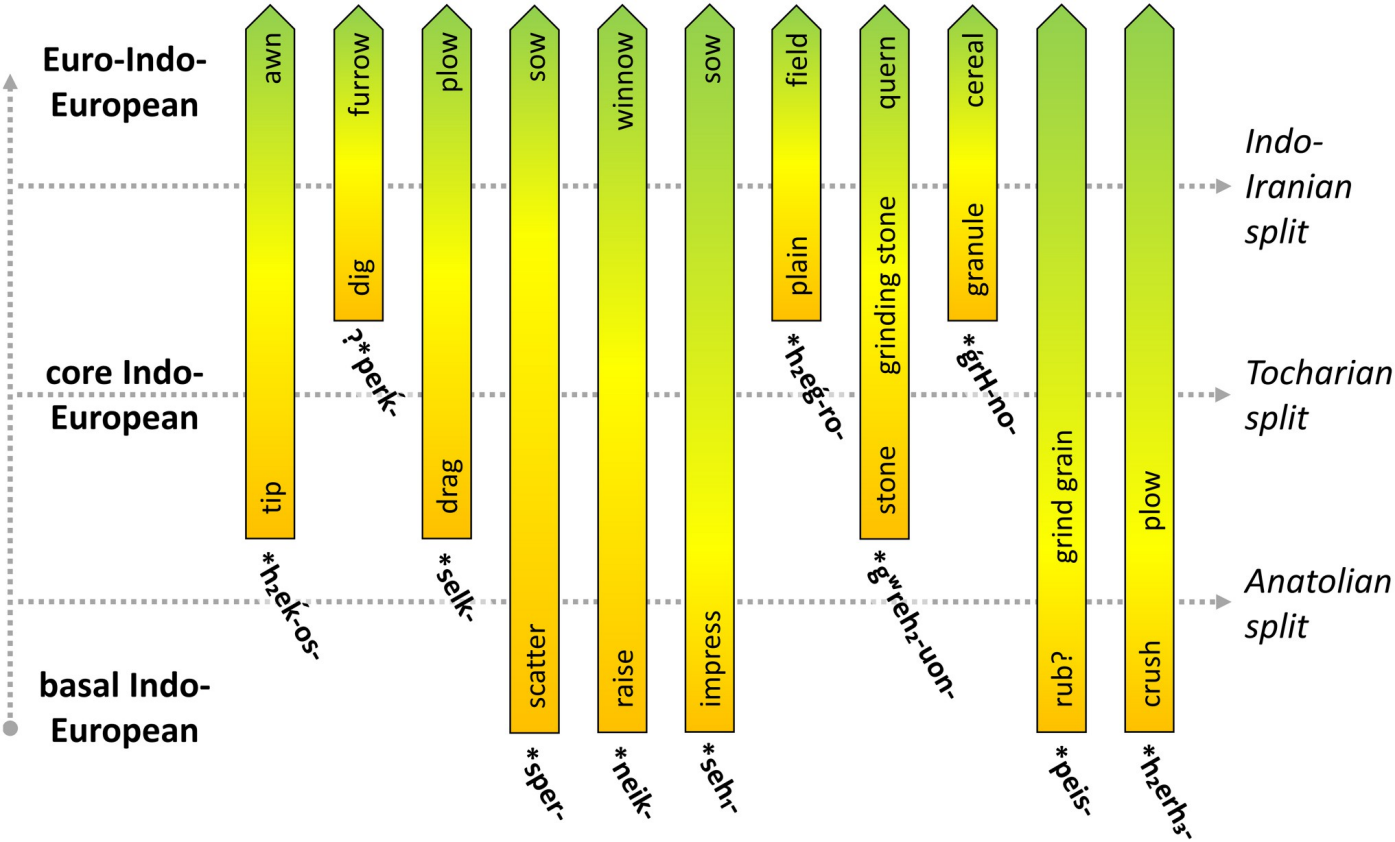
The emergence of cereal cultivation and processing terminology between Indo-Anatolian and the fragmenting core Indo-European dialect continuum. The reconstructed protoforms of some agricultural terms are placed in the figure to indicate in which phase of the Indo-European language family they emerged. The accompanying arrows show the evolution of the meanings of these protoforms through time.

Consequently, we may conclude that the evidence presented here is more consistent with Schrader’s scenario than with that of Hirt. While it cannot be excluded that Indo-Iranian lost some vocabulary, the data strongly suggest that the relative dearth of inherited agricultural terminology in this branch is due to a comparatively limited involvement in the lexical innovations that characterize the European branches. At the same time, it is clear that some vocabulary was lost in Indo-Iranian. As the root *h_*2*_*erh*_*3*_- is also attested with the meaning ‘plow’ in Tocharian, which is widely held to have split off second, Indo-Iranian probably once possessed this verb, something that also follows from the preservation of the formation *h_*2*_*rh*_*3*_-*ur/n*- ‘(arable) field’ in this branch. It thus appears that both Schrader and Hirt were partially right. On the one hand, Indo-Iranian participated in the initial core Indo-European shift from a pastoralist to an agro-pastoralist economy, of which some elements later were lost. On the other hand, Indo-Iranian was peripheral to the more recent and more radical shift towards a farming economy, as reflected in the vocabularies of the European branches (cf. Fig 2).

## 5. Discussion

The results from the present investigation mitigate, but do not entirely resolve the archaeolinguistic paradox outlined in the introduction. Through the lexical evidence, a cultural shift is observed from a presumably mobile, predominantly non-agricultural to a more sedentary, agro-pastoral language community. The former is represented by basal Indo-European, i.e. Indo-Anatolian, and the latter by core Indo-European, including Tocharian. A later, more radical shift towards an agricultural economy is seen in the European branches of the Indo-European family, which separated them from Indo-Iranian. Paradoxically, while the Yamnaya expansion offers the most plausible genetic vector for the spread of the core Indo-European languages from the Pontic Region, the archaeologically inferred economy of the Yamnaya populations between the Don and Volga rivers does not offer a perfect match for the linguistically inferred economy of the core Indo-European language community. Similarly, the closely related Afanasievo culture, with its lack of evidence for agriculture, does not provide an evidently suitable context for the Tocharian homeland. The question therefore is whether it is possible to identify an archaeological scenario that can more satisfactorily account for the transformation that took place between basal and core Indo-European, but without abandoning the connection with the population movements associated with the Yamnaya expansion.

The Indo-Anatolian phase does not in any way appear to be compatible with a fully-fledged agricultural lifestyle, as only one, perhaps two cereal terms can be reconstructed. Since familiarity with cereals does not necessarily imply familiarity with cultivation, and could also reflect trade or bartering [37:244], most of the Eneolithic cultures from the steppe and forest-steppe zone can be considered possible matches for the Indo-Anatolian speech community. Exchange may have happened through contacts with the Cucuteni-Trypillia culture (5200–2800 BCE) in the west or (the precursors of) the Maykop culture (3700–3000 BCE) in the east. A male from Dereivka dated to the early 5th millennium BCE genetically clusters with Trypillian farmers from Bulgaria [216; 217:329], demonstrating early contacts between the cultures. The first possible evidence for cultivation indeed comes from the Sredni Stog culture [218; 219]. The Dereivka and Molyukhov Bugor settlements appear to have supplemented their mainly hunter-herder-fisher subsistence with a hoe-based type of agriculture adopted from the west. Along the Lower Don, few cereal impressions are found, alongside chaff temper, in pottery of Rakushechny Yar and Zanovskoe [219]. However, cereals, either wild or domesticated, still played a marginal role in the diet of Eneolithic steppe groups, as confirmed by the absence of dental caries in a Sredni Stog individual [220:266]. The Sredni Stog has previously been connected with the Indo-Anatolian phase [3:262; 23], and the Anatolian split with the movements of the Suvorovo-Novodanilovka chiefs into the Balkans.

Much more than superficial knowledge of cereal use must be assumed for the later phases of the language family, even before the Tocharian split. This makes the eastern Yamnaya culture a less attractive archaeological fit for core Indo-European. Lexically, the transition from basal to core Indo-European resembles a language community penetrating a fundamental cultural barrier separating the pastoral and agricultural realms. Such a barrier has been identified archaeologically in the steppe as the Dnieper river, which, after the expansion of Trypillian farmers into the territories of the Bug-Dnieper culture, had functioned as a cultural border with non-agrarian societies for no less than two millennia [3:166, 264; 221:239–40]. This barrier was eventually shattered when steppe pastoralists became fully mobile, an event that appears fundamental to understanding the linguistic evolution of basal to core Indo-European.

**Fig 3:**
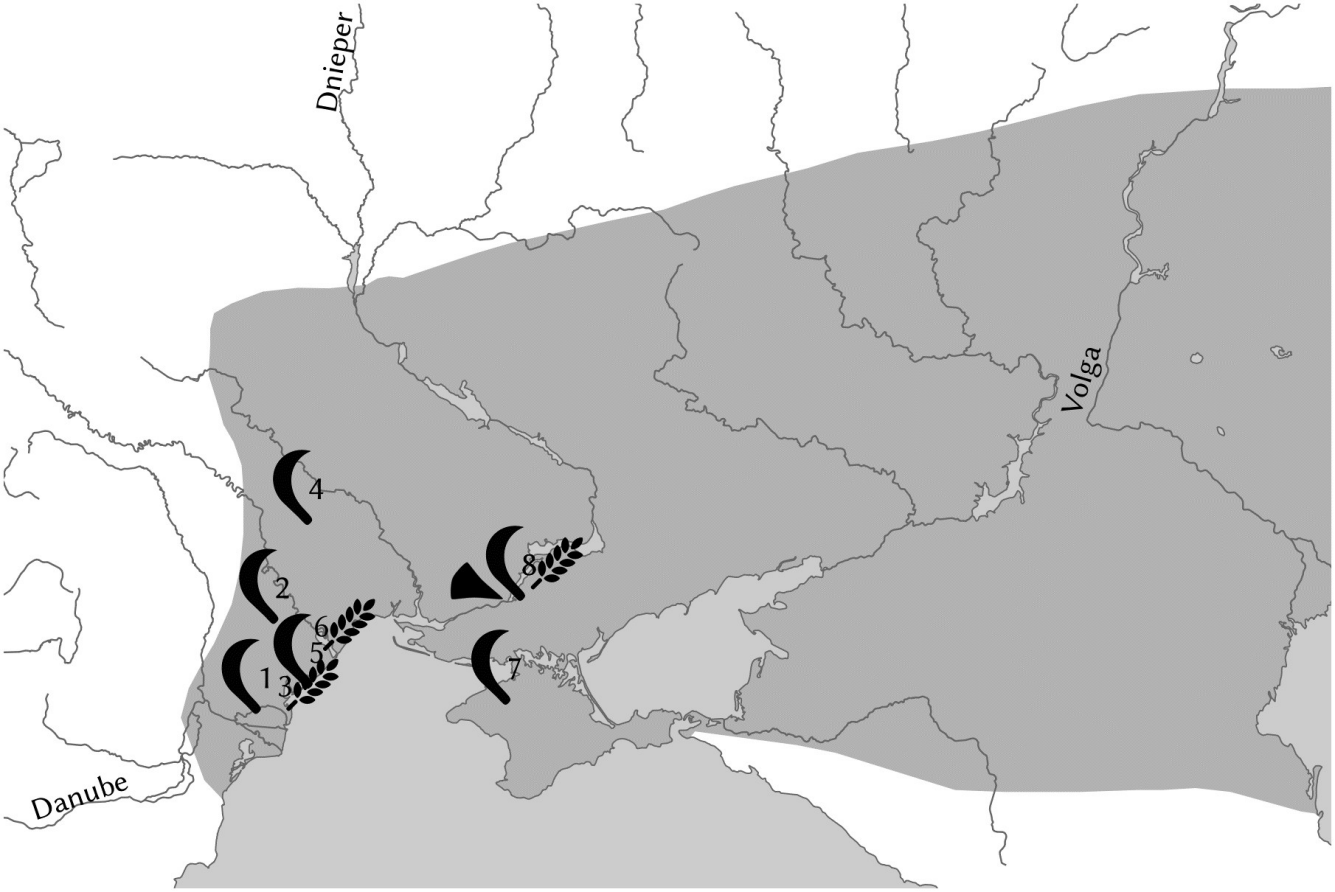
Cereal remains, cutting tools and a plowshare alleged to be found in Yamnaya contexts. The shaded area indicates the extent of the Yamnaya culture at the end of the Copper Age [18:651]. Sites: 1 Kholmske; 2 Gura-Bykuluy; 3 Glubokoe; 4 Tetskany; 5 Alkaliya; 6 Belyaevka; 7 Rysove; 8 Mikhailovka; 9 Skelya-Kamenolomnya. Cutting tool data from Razumov [162] and Ivanova [39], plowshare data from Gimbutas [214:161], and cereal data from Pashkevich [36:15] and Anthony [3:320].

Around 3400 BCE, the transition from Phase I to Phase II of the Mikhailovka settlement, located on the western bank of the Lower Dnieper, marks a shift from farming to cattle herding and the introduction of Repin-style pottery [3:320–1]. The evidence for farming does not disappear, however, and Mikhailovka II/III appears to have been a settled Yamnaya site whose inhabitants practiced sporadic agriculture [28; 32:904]. From 3300, Yamnaya pastoralists crossed the Dnieper in increased numbers and started settling the westernmost steppes. At the same time, Late Cucuteni-Trypillian farmers were expanding into the steppe directly west of the Middle Dnieper, where settlements persisted until 2600 BCE, resulting in a short-lived but likely crucial phase of coexistence in this area [2:237; 222]. Kurgans were erected on top of Late Cucuteni settlements [223:301; 224]. Cereal imprints are documented for two of the Belyaevka and Glubokoe kurgans on the lower Dniester [3:320]. Further west in the Lower Danube region, regionally distinguishable burial customs reflect the adaptation of incoming pastoralists to the local populations of the tell settlements [225]. Within a few generations, culturally and linguistically diversifying Yamnaya groups would have had ample opportunity to acquire extensive knowledge of local agricultural practices, such as the use of plows, plowshares and sickles, as they have been documented archaeologically in the region in the fourth millennium BCE [43; 127:88–95; 162:48; 212], as indicated in Fig 3.

In conclusion, unlike the archaeological Yamnaya homeland, the linguistic homeland of the core Indo-European language community cannot be located in the eastern steppe, but must be situated around, and extending to the west of, the Dnieper River. After the formation of the core Indo-European dialect continuum in this area after ca. 3300 BCE, it gradually developed into a network of increasingly evolved and disconnected varieties of Indo-European speech, thus foreshadowing the final fragmentation of the language and the movements of the various branches into Europe and Asia. Intriguingly, Indo-Iranian and especially Tocharian were impacted less heavily by the later, more radical shift towards agriculture that manifests itself in the European branches, indicating that they were culturally but also geographically more peripheral. However, since these branches share the Indo-European words for ‘plow’ and ‘pound grain’, they must, too, somehow have been involved in or at least connected to the establishment of the core Indo-European continuum in the West Pontic region. Scenarios in which the European branches moved west and the Asian branch stayed east of the Dnieper [226] therefore appear overly simplistic. While Gimbutas was largely correct in assuming that “the increase of agriculture is synchronous with the incursion of the Kurgan […] people into Europe” [227:395], especially in the European branches, we must assume that the onset of this process had already started before the final dissolution of the core Indo-European dialect continuum, on or close to the steppe. Quite possibly, segments of the core Indo-European speech community moved west before they moved east, including those groups that ultimately introduced Tocharian and Indo-Iranian to Asia. For the steppe component in Indo-Iranians, the Eastern European Corded Ware has been suggested as the mediator of Yamnaya ancestry [228]. For Tocharian, it may be necessary to assume an indirect dispersal as well in view of the late spread of agriculture to the eastern steppe. The wooden plows of the Catacomb culture (2500–1950 BCE) offer an archaeological *terminus post quem*. A successive potential proxy is the Babyno culture (2200–1700 BCE), whose similarities to the Epi-Corded Ware of the Carpathian region suggest an East-Central European origin [229].

A central question concerns the mechanism by which mobile pastoralism was adopted in the Lower Dnieper region during the westward expansion of the Yamnaya culture. Did incoming herders displace local groups, including their language, before the final expansion into Europe and Asia? Or did local groups adopt this lifestyle purely culturally, subsequently to become the source population that ultimately proliferated its genetic and linguistic features to much of Eurasia? From the linguistic perspective, it is worth noting that the Sredni Stog culture, with its limited evidence for agriculture, potentially offers a better archaeological fit for the basal, Indo-Anatolian language community than the eastern Yamnaya culture, which shows no traces of agriculture. This may support a scenario of linguistic continuity of local non-mobile herders in the Lower Dnieper region and their genetic persistence after their integration into the successive and expansive Yamnaya horizon.

These archaeolinguistic considerations may furthermore help shed light on the genetic origins of the Yamnaya dispersal. The Corded Ware and Bell Beaker cultures, both promising vectors of Indo-European speech varieties, have high levels of steppe ancestry [6–8], but due to a mismatch in the Y-haplogroups, the exact genetic source population that contributed to the Corded Ware so far remains elusive [230:386–95; 231]. The evolution of the Indo-European lexicon implies that cattle breeders interacted closely with contemporaneous farmers in the Northwest Pontic Region prior to the linguistic dispersals of the majority of Indo-European subgroups. Arguably, the linguistic interactions between farmers and pastoralists resulted from some of the same processes that contributed to the emergence of the major archaeological complexes that soon came to dominate much of Late Neolithic Europe [232]. In-so-far as linguistic evidence can be employed to elucidate human genomic prehistory, the reconstructed vocabulary of core Indo-European culture suggests that the source populations for the steppe ancestry in the earliest Bell Beaker and Corded Ware groups should be sought in the Pontic rather than the Caspian steppe and forest-steppe zones.

## Abbreviations

Akk.: Akkadian
Alb.: Albanian
An.: Anatolian
Arm.: Armenian
Att.: Attic
Av.: Avestan
Bact.: Bactrian
Bav.: Bavarian
Biel.: Bielorussian
Bret.: Breton
BSl.: Balto-Slavic
C: Celtic
Cat: Catalan
Chuv: Chuvash
Corn: Cornish
Cr.: Croatian
CS: Church Slavonic
Cypr: Cypriot
Cz: Czech
D: Digor
Da.: Danish
Dor.: Doric
Du.: Dutch
E: English
Est.: Estonian
Far.: Faroese
Fi.: Finnish
Fr.: French
G: German
Gaul.: Gaulish
Ge.: Georgian
Gk.: Greek
Go.: Gothic
Hitt.: Hittite
Hung.: Hungarian
Hur.: Hurrian
I: Iron
Icel.: Icelandic
IE: Indo-European
Ir.: Iranian
It.: Italic
Ital.: Italian
Jass.: Jassic
Kal.: Kalasha
Khot.: Khotanese
Khow.: Khowar
Ko.: Komi
Lac.: Laconian
Lat.: Latin
Latv.: Latvian
Lith.: Lithuanian
Liv.: Livonian
LLat.: Late Latin
Luw.: Luwian
Ma.: Mari
MBret.: Middle Breton
MDu.: Middle Dutch
ME: Middle English
MHG: Middle High German
MIr.: Middle Irish
MoBret.: Modern Breton
Mong.: Mongolian
MP: Middle Persian
Mrd.: Mordvin
MW: Middle Welsh
Norm.: Norman
NP: New Persian
Nw.: Norwegian
OBret.: Old Breton
OCo.: Old Cornish
OCS: Old Church Slavonic
OE: Old English
OFr.: Old French
OFri.: Old Frisian
OGutn.: Old Gutnish
OHG: Old High German
OIr.: Old Irish
ON: Old Norse
OProv.: Old Provençal
OPru.: Old Prussian
ORu.: Old Russian
OS: Old Saxon
Oss.: Ossetic
OSw.: Old Swedish
OW: Old Welsh
P: Persian
Pa.: Pali
PAlb.: Proto-Albanian
PAn.: Proto-Anatolian
PArm.: Proto-Armenian
Parth.: Parthian
PB: Proto-Baltic
PBr.: Proto-Brittonic
PBSl.: Proto-Balto-Slavic
PC: Proto-Celtic
PEB: Proto-East-Baltic
PGk.: Proto-Greek
PGm.: Proto-Germanic
PIE: Proto-Indo-European
PIIr.: Proto-Indo-Iranian
PIr.: Proto-Iranian
PIt.: Proto-Italic
Pk.: Prakrit
Pol.: Polish
PRom.: Proto-Romance
Psht.: Pashto
PSl.: Proto-Slavic
PTo.: Proto-Tocharian
PWB: Proto-West-Baltic
Rom.: Romanian
Rosh.: Roshani
Ru.: Russian
Sab.: Sabellic
SCr.: Serbo-Croatian
Shu.: Shughni
Skt.: Sanskrit
Sl.: Slavic
Sln.: Slovene
Sogd.: Sogdian
Sp.: Spanish
Sw.: Swedish
Taj.: Tajik
To.: Tocharian
ToB: Tocharian B
Udm.: Udmurt
Ukr.: Ukrainian
Umbr.: Umbrian
Val.: Valencian
Ved.: Vedic
W: Welsh
Wakh.: Wakhi
WFri.: West Frisian
Wj.: Wanji
WSem.: West Semitic
YAv.: Young Avestan
Yazg.: Yazghulami
Yd.: Yidgha
